# Retracing the Response of Rangifer to Postglacial Climate Change in Arctic Islands

**DOI:** 10.1002/ece3.73125

**Published:** 2026-03-19

**Authors:** Maria Dance, Erin E. Saupe, Alex Farnsworth, Paul J. Valdes, Marc Macias‐Fauria

**Affiliations:** ^1^ School of Geography and the Environment University of Oxford Oxford UK; ^2^ Scott Polar Research Institute University of Cambridge Cambridge UK; ^3^ Department of Earth Sciences University of Oxford Oxford UK; ^4^ School of Geographical Sciences University of Bristol Bristol UK

**Keywords:** approximate Bayesian computation, caribou, phylogeography, postglacial dispersal, *Rangifer tarandus*, reindeer, sea ice

## Abstract

*Rangifer tarandus* L. plays a key role in Arctic ecosystems as the most numerous and widespread large herbivore. Sea ice is vital for maintaining genetic connectivity in Arctic islands, yet the historical role of sea ice in shaping *R. tarandus* biogeography is unknown. We studied the role of sea ice changes and ice sheet retreat since the last glacial period in the timing of island dispersal. We compiled published datasets of mitochondrial control region sequences that informed population history scenarios, which were evaluated in a coalescent‐based approximate Bayesian computation (ABC) modelling framework to test hypotheses of island (re)colonisation and to estimate divergence and admixture. Population events were compared with modelled and proxy‐based paleo‐sea ice cover and published ice sheet chronologies. Our analysis supports Holocene dispersal onto deglaciated Arctic islands, rather than High Arctic glacial refugia. The degree of population admixture and the effect of sea ice were dependent on regional geography and climate history. North American initial island population divergence occurred as sea ice cover was declining. A lack of strong genetic structure and the occurrence of late Holocene admixture suggest that Canadian Arctic Archipelago populations were somewhat connected by sea ice during the Holocene. The Svalbard, Franz Josef land, and West Greenland colonisations arose through long‐distance dispersal. Here, divergence times occurred post‐deglaciation but broadly align with subfossil‐based colonisation estimates, suggesting dispersal limitation due to sea ice conditions, potentially requiring appropriate ocean currents and sea ice drift directionality and speeds. Our study sheds light on the Late Quaternary (~60 ka—present) history of Arctic island *Rangifer* and suggests that ice sheet retreat, sea ice, and ocean currents were important in shaping present‐day genetic patterns. Regional differences in postglacial dynamics suggest that dispersal during contemporary climate change may vary regionally and depend upon diminishing connectivity provided by sea ice.

## Graphical abstract

Our study examines the role of sea ice and glacial retreat in shaping the biogeography of *Rangifer tarandus* across Arctic islands since the last glacial period. Using mitochondrial DNA analysis and approximate Bayesian computation (ABC) modelling, we found that Arctic island dispersal occurred during the Holocene, with sea ice cover likely influencing genetic connectivity regionally. The findings suggest that ongoing climate change may impact dispersal patterns due to reduced sea ice connectivity.

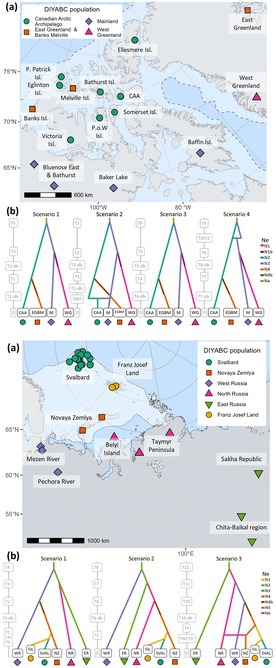

## 1 Introduction

*Rangifer tarandus* L., referred to as reindeer in Eurasia and caribou in North America (*Rangifer* hereafter), is the most numerous and widespread large herbivore in the Arctic, and therefore has large effects on other species, tundra vegetation composition, and ecosystem functioning (Olofsson and Post 2018). The species' range includes the High Arctic island systems of North America and the Barents and Kara Seas (COSEWIC 2011; Gunn et al. 2010; le Moullec et al. 2019; Mizin et al. 2018). The long‐term viability and genetic variation of these smaller, fragmented populations are reduced as a result of restricted gene flow, increased genetic drift, and inbreeding (Burnett et al. 2023; Kellner et al. 2024; Petersen et al. 2010; Schlaepfer et al. 2018; Taylor et al. 2024). Population connectivity is important in maintaining population numbers, genetic variation, and subsequent adaptive capability on these islands in the face of environmental change (Mallory and Boyce 2018; Torda and Quigley 2022).

In the Arctic, sea ice is vital for island colonisation and biological connectivity in *Rangifer* populations by enabling crossings between islands (Jenkins et al. 2016; Miller et al. 2005; Mizin et al. 2018; Peeters et al. 2019; Poole et al. 2010). Loss of population genetic connectivity following contemporary sea ice decline has been documented in both the Canadian Arctic and Svalbard archipelagos, where continued sea ice loss is expected (Jenkins et al. 2018; Mallory and Boyce 2018; Peeters et al. 2019).

Regional differences in connectivity exist among Arctic island *Rangifer* populations because of varying geographical distance between islands, distance from the mainland, and sea ice conditions (including dominant ocean surface currents that are key in ice drift direction and velocity). In the Canadian Arctic Archipelago (CAA), *Rangifer* regularly cross over sea ice as part of seasonal migrations and sporadic dispersal events (Miller et al. 2005; Poole et al. 2010). In the CAA, inter‐island distances can be as narrow as 2–20 km, and sea ice cover has been consistently present on a seasonal basis throughout the Holocene (Bobylev and Miles 2020; Briner et al. 2016). Crossings between the CAA and Greenland are much rarer, although documented (COSWIC 2015; Taylor 2005), despite the two regions being separated only by the Nares Strait (40 km wide at its narrowest point) and connected annually by sea ice bridges (Moore et al. 2021). Northwest Greenland's marine‐terminating glaciers further isolate the ice‐free west of Greenland from the CAA.

*Rangifer* population genetic connectivity within the Svalbard archipelago also depends on sea ice (Peeters et al. 2019), even though sea ice is more variable in time and space than in the CAA due to warm Atlantic water inflow from the West Spitsbergen Current. Large distances between land masses in the Barents Sea further limit opportunities for gene flow between the region's archipelagos and the mainland. Svalbard lies approximately 400 km from the Franz Josef Land archipelago and 770 km from Novaya Zemlya (itself separated from the Russian mainland by the 56 km wide Kara Strait). Only a few sightings of *Rangifer* migrating between the mainland and Barents Sea islands have ever been recorded (Hoel 1916; Lønø 1959). Favourable sea ice drift direction and speed may also be important factors for sea‐ice‐mediated connectivity, particularly for long‐distance dispersal. Ocean currents move sea ice up to several kilometres per day (Kaur et al. 2018), and so can either increase the effective distance that animals travelling in the same direction can cover with no additional energetic cost, or do the opposite if animals travel against the direction of the ice drift. Determining how historical changes in sea ice have influenced dispersal and connectivity in Arctic archipelagos is critical for understanding the evolutionary consequences of continued sea ice loss. Changes in connectivity due to sea ice changes are detectable in contemporary geographic patterns of genetic diversity (Mellows et al. 2012; Norén et al. 2009). However, genetic data have not been used to assess the historical role of sea ice in enabling *Rangifer* dispersal to and between Arctic islands, and the colonisation history and evolutionary relationships remain unclear (Mizin et al. 2018).

The taxonomic and evolutionary relationships of Arctic island *Rangifer* populations are complex. Arctic island Rangifer occur in small‐bodied, large‐bodied, and intermediate forms, consisting of several subspecies, of which mainly the small‐bodied subspecies occur in Arctic islands, in agreement with the island rule (Benítez‐López et al. 2021; Lomolino et al. 2013). The Svalbard ( *R. tarandus platyrhynchus* ) and North American (R. *t. pearyi*) small‐bodied subspecies were thought to have a monophyletic origin (Hakala et al. 1986; Røed et al. 1986), but mitochondrial sequences suggest that both subspecies evolved convergently from different source populations: *R. t. pearyi* from the Euro‐Beringian lineage in North America, and *R. t. platyrhynchus* from the Euro‐Beringian lineage in Eurasia (Gravlund et al. 1998; Hold et al. 2024; Taylor et al. 2024). The Euro‐Beringian lineage likely spent the last glacial period in ice‐free parts of Eurasia, including the ice‐free Beringian region that spanned from the Verkhoyansk mountains in Eastern Russia to the Mackenzie River in North America (Elias and Brigham‐Grette 2013; Hultén 1937).

Svalbard *Rangifer* likely originated from the Russian mainland and reached the archipelago via Novaya Zemlya and Franz Josef Land, evidenced by strong genetic connections (Dussex et al. 2023; Hold et al. 2024; Kvie et al. 2016). North American small‐bodied *Rangifer* have been proposed to have either (i) colonised islands in the postglacial period (Flagstad and Røed 2003) or (ii) been isolated in a separate, ice‐free High Arctic refugium and then mixed with mainland *Rangifer* in the postglacial period (Eger et al. 2009; Klütsch et al. 2017).

Constrained time estimates of island colonisation are needed to determine the potential roles of Quaternary ice sheet and sea ice changes in shaping *Rangifer* population history. Estimates of population divergence and admixture times from coalescent models, which link extant genetic diversity with population demographic history (Kingman 1982; Marchi et al. 2021), can help infer the biogeographic barriers and corridors that have shaped species' Late Quaternary histories (Hansen et al. 2018; Robinson et al. 2014; Sousa et al. 2012). Such methods have been used previously to infer population histories and impacts of Quaternary climate change on *Rangifer* (Eger et al. 2009; Klütsch et al. 2017; Lorenzen et al. 2011; Polfus et al. 2017; Taylor et al. 2021; Yannic et al. 2014), including *Rangifer* populations in the CAA (Klütsch et al. 2017). Recent studies using whole genomes have also investigated postglacial colonisation routes and timings in the Barents Sea islands (Dussex et al. 2023; Hold et al. 2024). However, an inter‐regional comparison of *Rangifer* population history and sea ice dynamics has not yet been made.

Here we reconstructed and compared *Rangifer* population histories in two Arctic island systems (North American Arctic islands, NAAI and Barents Sea islands, BSI) using mitochondrial control region sequences and approximate Bayesian computation (ABC). We compare evolutionary scenarios to test whether island populations (i) shared a common location with mainland populations during the Last Glacial Maximum (LGM) and dispersed postglacially, or (ii) were isolated prior to the LGM in separate High Arctic refugia and met mainland populations during the LGM or the postglacial period. Using the best‐supported scenarios, we compare timings of population divergence and admixture with modelled sea ice cover, proxy‐based sea ice reconstructions, and ice sheet retreat to infer the likely drivers of *Rangifer* dispersal.

We hypothesised that island colonisation coincided with the emergence of newly‐available land after ice sheet retreat, providing that sea ice cover was sufficient (> 70% cover) for dispersal. We further hypothesised that the colonisation of the two island systems differed because of varying geography, deglaciation patterns, and sea ice history. Specifically, in the NAAI we expected that more secondary contact and population admixture occurred in periods of sufficient sea ice cover. We expected NAAI population divergences to have occurred during reduced sea ice cover, as populations previously connected became isolated. In the BSI, we expected stepwise dispersal with fewer opportunities for secondary contact. Divergence times should correspond to island colonisation as founding populations were separated from source populations by long‐distance dispersal over sea ice. Population divergence is therefore expected to be associated with sufficient sea ice cover in the Barents Sea. Testing these hypotheses is important for developing our understanding of the long‐term ecological and evolutionary impacts of past climate change on *Rangifer*, and the potential impacts of an increasingly ice‐free Arctic on the population connectivity and long‐term persistence of ice‐influenced species.

## 2 Materials and Methods

### 2.1 Species

Several recognised subspecies of *R. tarandus* inhabit the Arctic islands. The two extant small‐bodied species, *R. tarandus platyrhynchus* (Vrolik, 1829) and *R. t. pearyi* (Allen, 1902) occur in Svalbard and in the Canadian Arctic Archipelago (CAA), respectively. Small‐bodied *Rangifer* described as *R. t. eogroenlandicus* (Degerbøl 1957; Meldgaard 1986) inhabited East Greenland but were extirpated around 1900 and were considered genetically and morphologically close to *R. t. pearyi* (Gravlund et al. 1998). *Rangifer t. platyrhynchus* and *R. t. pearyi* are specialised for the High Arctic environment and share similar morphologies of small body size, short legs and muzzle, and paler, longer winter fur than their mainland neighbours.

In North America, large‐bodied caribou (*R. t. groenlandicus*) occur on Baffin Island and West Greenland, as well as on the continental tundra (COSEWIC 2011; McFarlane et al. 2016; Meldgaard 1986). The Dolphin‐Union herd in the CAA is currently designated as an intermediate form *R. t. groenlandicus* x *pearyi* (COSEWIC 2011). In the Eurasian Arctic, large‐bodied *Rangifer* on Novaya Zemlya are variously recognised as the subspecies *R. t. pearsoni* (Lydekker, 1903; Kvie et al. 2016; Mizin et al. 2018). *Rangifer* were also formerly present on the Franz Josef Land archipelago during the Holocene, with subfossils dated to between 6.4 to 1.3 ka (Forman et al. 2000).

### 2.2 Genetic Data

We used 524 mitochondrial control region sequences from previously‐published datasets, details of which are outlined in Table A1.1, augmented with 128 recently published mitogenomes, trimmed to match the length of the CR sequences (Hold et al. 2024). In *Rangifer*, the rapidly evolving mitochondrial control region has been found to be suitable for exploring intraspecific genetic relationships (Eger et al. 2009; Gravlund et al. 1998). Sequences were obtained from NCBI GenBank (Sayers et al. 2022), and haplotype‐level sequences were converted to individual‐level sequences with a corresponding population identification using study metadata. Sequences from domesticated herds and potentially domestically‐introgressed wild populations were excluded, except for the key population of Novaya Zemlya, where possible introgression from domestic *Rangifer* has been suggested (Mizin et al. 2018). DNA from the extinct East Greenland *Rangifer* species (*R. t. oegroenlandicus*) was included in this study, with samples collected in 1892 (Gravlund et al. 1998). The software MAFFT v 7.526 (Katoh and Standley 2013) with the option –add was used to align the mitochondrial CR sequences to the published mitogenome alignment.

All further analyses were run using R v 4.4.3 (R Development Core Team 2024) in R studio v 2024.12.1 (Posit team 2024), unless otherwise stated. We divided genetic data into two geographic regions, the North American Arctic islands (NAAI) and the Barents Sea islands (BSI), before trimming to maximise sequence lengths within each region. Sequences were trimmed to the same length to minimise problems with lower quality read ends, remove missing data, and because ABC software cannot handle indel mutations (Collin et al. 2021). Basic genetic summaries and pairwise *F*_ST_ measures (Nei's Gst, Hedrick's G'st, and Jost's D) were calculated using hierfstat v 0.5–11 and mmod v 1.3.3 packages, respectively (Goudet et al. 2022; Winter 2012). Haplotypes were extracted using the pegas v 1.3 package (Paradis 2010).

Population and individual sampling locations were inferred where coordinates were not readily available using locality metadata and georeferenced using the Google Geocoding API in the ggmap v 4.0.1 package (Kahle and Wickham 2013) or obtained from online sources (Alaska Center for Conservation Science 2019; Environment and Climate Change, Government of the Northwest Territories 2023). Sequences from eastern Russia were included in the BSI dataset to test whether the Svalbard population arose from the Beringean glacial lineage or whether it diverged earlier in a separate glacial location (Kvie et al. 2016). The North American lineage—which occurred south of the North American ice sheets during the LGM—was excluded from the analysis since the large genetic distance it showed from the Beringean group made it difficult to distinguish fine‐scale regional phylogeographic patterns (Kuhn et al. 2010; Yannic et al. 2014).

### 2.3 Population Genetic Structure

Each individual genetic sample had been associated previously with a population, herd, or locality based on sampling location. To visualise the multivariate genetic dissimilarity between original populations, we used metric multidimensional scaling (mMDS) of genetic distance matrices within the cmdscale function (R Development Core Team 2024). To determine the number of distinct genetic populations, we used exploratory clustering approaches without assumptions on the underlying evolutionary model (Jombart et al. 2009). Non‐evolutionary Euclidean distance matrices were calculated using the adegenet v 2.1.11 R package (Edward's distance) (Jombart 2008) and dendrograms constructed using the unweighted pair group method with arithmetic mean (UPGMA) hierarchical clustering, implemented with the hclust function (R Development Core Team 2024). Various agglomeration methods were tested and the method with the highest correlation between original distances and resulting tree‐based distances chosen (“average method”). UPGMA dendrograms were plotted using the dendextend v 1.19.0 package (Galili 2015).

*K*‐means clustering was performed on both the genetic distance matrices and the mMDS dissimilarity coordinates and overlaid on (a) the UPGMA dendrogram to compare groupings obtained by the different clustering methods (Galili 2015), and (b) the mMDS ordination plot to identify genetic clusters. *K*‐means clustering assigned each observation to the cluster with the nearest mean. The optimal number of clusters (*K*) was estimated by both (i) examining plots of within‐group variance at each value of *K* and selecting the highest value of *K* beyond which the variance did not markedly decline (Elbow method), and (ii) determining how well each object lies within its cluster for different values of K, using the average silhouette method in the cluster v 2.1.8 package (Maechler et al. 2022).

### 2.4 Development of Demographic Modelling Scenarios

We used the clustering methods to inform population groups and relationships in evolutionary scenarios that represented different hypotheses of population history. Initial pre‐selection of plausible evolutionary scenarios is practical, given the prohibitively high number of potential demographic scenarios for multiple populations and the risk of multiple testing or overfitting when performing many analyses or testing complex models (Borrell et al. 2018). Seventeen historical and ancient Svalbard samples were included in the population clustering analyses as they form a single lineage with the modern samples (Kellner et al. 2024) but were excluded from demographic modelling as all samples within a population needed to be sampled at the same time in DIYABC‐RF (Collin et al. 2021). Three ancient Franz Josef Land samples with median radiocarbon ages ranging from 2.7 to 4.7 ka were included in the demographic modelling as their own population. A weighted mean sample age of 502 generations for Franz Josef Land was calculated using the sample median radiocarbon ages and their standard deviations divided by the generation time of 7 years (Klütsch et al. 2017; Polfus et al. 2017).

For each scenario, the following parameters were specified: (i) population groups, (ii) topology of the evolutionary scenario and corresponding sequence of population events (population divergence, admixture, and bottlenecks), (iii) prior time ranges for population events, (iv) prior ranges for the effective population sizes (Ne) of modern populations and bottleneck magnitude, and (v) genetic data characteristics, including mutation rate and mutation model.

We grouped evolutionary scenarios into sets of models that could be tested against each other because they had the same population groups. Within each model set, scenarios differed in their topology or parameter prior ranges. The optimal number of genetic clusters identified in *K*‐means clustering was used as a starting point for the population groupings. As the model sets were tested, new model sets were made where populations were split further or combined based on F statistics to minimise within‐population genetic structure and maximise between‐population differences.

Scenarios of a single glacial population with postglacial divergence were tested against scenarios with earlier divergence and separate glacial locations (Klütsch et al. 2017; Kvie et al. 2016). For the NAAI, we also tested a scenario of island population divergence between 100 and 27 ka, admixture with the mainland in the Last Glacial Maximum, followed by postglacial dispersal. We developed and tested 35 scenarios grouped in 10 model sets for the NAAI and 30 scenarios grouped in 17 model sets for the BSI. See Tables A2.1–A2.5 for details on model sets, scenarios, and their prior specifications.

### 2.5 Testing Evolutionary Scenarios

We used approximate Bayesian computation (ABC) to test our evolutionary scenarios (Beaumont 2010; Cornuet et al. 2014). Within each model set, genetic data were simulated under the different evolutionary scenarios using a population genetic simulator, summarised by a large set of statistics, which were then compared to the observed genetic data statistics. The software DIYABC RF was used for ABC analyses in combination with supervised machine learning based on Random Forests (RF) (Collin et al. 2021) for training set simulation, model selection and parameter estimation. DIYABC RF was implemented using the command‐line software DIYABC RF v1.1.27 and abcranger v1.16.30 for linux. Using RF models is computationally efficient, addresses potential ABC limitations, and outperforms other ABC methods for analysis of multiple complex scenarios (Collin et al. 2021). For each model set, we generated 100,000 simulated genetic datasets, drawing parameter values from the prior distributions. The number of trees for model selection and parameter estimation was fixed to 500 to ensure stable estimation of the global error rate. Otherwise, default software parameters were used.

For each model set, we assessed overlap between 1000 randomly selected genetic datasets simulated under each scenario with the observed genetic dataset on the first two axes of a principal component analysis (PCA). Visual assessment of dataset overlap identified poorly specified scenarios and helped to refine prior ranges for new model sets. When observed and simulated datasets overlapped, scenarios were tested against each other using RF model classification. The best‐performing models were selected based on model error rates and on visual assessment of overlap between simulated and observed data when projected on linear discriminant analysis (LDA) axes. LDA also indicated the degree to which it was possible for RF classification to discriminate among scenarios within a model set.

The best‐performing scenarios were iteratively improved and tested until a final set of models was produced in which local model error rate was reduced to approximately 20% or less (Table 1 in Collin et al. 2021), complete overlap occurred between simulated and observed data, and posterior probabilities were above 65% (Collin et al. 2021). See Tables A2.4 and A2.5 for detailed DIYABC specifications of the scenarios in the final model set.

### 2.6 Population History Parameter Estimation

The most plausible evolutionary scenario with the highest posterior probability was selected from each final model set, and divergence, admixture, and bottleneck time parameters were estimated. We conducted independent RF models for each parameter of interest. Point estimates and 95% credible intervals were calculated along with global and local accuracy metrics. Estimates were converted from number of generations to calendar years assuming a generation time of 7 years (Klütsch et al. 2017; Polfus et al. 2017), although some other studies have assumed a generation time of six years (Dussex et al. 2023; Kellner et al. 2024).

### 2.7 Comparison With Paleo Sea Ice Data

We compared estimated population event times from the preferred models with modelled sea ice concentration to assess whether estimated times of divergence and admixture corresponded to changes in sea ice cover in regions that may have supported *Rangifer* crossings.

Paleo‐sea ice concentration was estimated from simulations using the global HadCM3B coupled atmosphere–ocean general circulation model with dynamic paleovegetation (specifically HadCM3LB‐M2.1aD; Singarayer et al. 2017; Valdes et al. 2017). The model includes ice sheet evolution and continental, isostatic rebound, from which ice sheet extent and a land sea mask for each interval were obtained. Therefore, an indication of changes in sea level through time and land bridges as potential dispersal routes could be assessed. A detailed description of HadCM3LB‐M2.1aD and the sea ice component model is available in Valdes et al. (2017). When compared with observational datasets, the model accurately represents many aspects of the climate system on global and regional scales, including sea surface temperatures and ocean circulation (Valdes et al. 2017). For the paleoclimate simulations, sea‐ice cover compares well with estimates of ice extent from microfossil data in regions with available data (Roche et al. 2012; Singarayer and Valdes 2010). Further information on the model can be found in Appendix S3.

In total, 25 individual simulations covering the last 24,000 years were run at a time resolution of 1000 years. Each simulation was run for 500 model years, with the last 50 years used to compute climate means.

Sea ice concentration was summarised as monthly and annual climatological means for discrete snapshot simulations at the highest available resolution of one thousand years. Large sea ice changes can occur on sub‐millennial timescales (Kinnard et al. 2011; Saini et al. 2022), but changes in sea ice that persist on millennial timescales are suitable for comparison with demographic history events, as it can take many generations for a change in population structure or connectivity to be evident in the genetic structure (Epps and Keyghobadi 2015). Furthermore, millennial‐scale increases in sea ice cover provide more opportunities for rare and random long‐distance dispersal of *Rangifer* to islands across sea ice (Gillespie et al. 2012; Jordano 2017). We focused on the Last Glacial Maximum to the preindustrial period (ca. 21–0 ka), as our best‐supported demographic models suggested postglacial dispersal to islands (see Results).

Estimated population event times were compared with monthly and annual mean sea ice concentrations at the closest corresponding climate model time step to determine temporal congruence with sea ice changes, and whether sea ice concentrations were sufficient to enable crossings (Mallory and Boyce 2018; Poole et al. 2010). To obtain estimates of sea ice concentration that were spatially relevant to the inferred population events, we extracted sea ice cover from geographical areas corresponding to likely *Rangifer* crossing regions. Each DIYABC population usually consisted of several herds and was therefore spread over space. To estimate the overall geographic position of these populations, we calculated the centroid between herds using the sf v 1.0–20 R package (Pebesma et al. 2025). Then, for each population event, we calculated geographic position as the centroid between populations involved in the event. To reduce the influence of selecting an arbitrary centroid, we sampled 100 random points within a 100 km radius of the centroid. For each of the 100 random points, we extracted sea ice concentration estimates from grid cells within a set radius: the NAAI model used 1000–1500 km radii to encompass all likely crossing regions within the Canadian Arctic Archipelago and Greenland. The BSI system had clearer potential crossing regions, so a 200 km radius was calculated for the Svalbard‐Franz Josef Land crossing region, a 300 km radius between Novaya Zemlya and Franz Joseph Land, and a 500 km radius between West Russia and Novaya Zemlya. Mean and standard deviation of sea ice concentration were summarised across grid cells within each radius. We projected sea ice and population data to the North Pole Lambert Azimuthal Equal Area projection (EPSG 3575) at 30 km resolution, centred at 100°W longitude for the NAAI model and 90° E longitude for the BSI model. Spatial data processing was conducted using the terra v 1.8–42 (Hijmans et al. 2025) and sf v 1.0–20 R packages (Pebesma et al. 2025).

Where available, published regional sea ice reconstructions derived from proxies were used as an independent comparison (and evaluation) to model data (Briner et al. 2016; Hörner et al. 2017; Jennings et al. 2011; Pieńkowski et al. 2013, 2021).

Finally, estimated divergence times were compared with the pattern and timing of ice sheet retreat over the relevant regions, using time slice reconstructions of ice margins at 1‐thousand‐year (ka) resolution based on recent syntheses of published dates from geological and geomorphological records (Dalton et al. 2020; Hughes et al. 2016; Winsborrow et al. 2023). Estimated sea levels further informed assessments of potential migration/refugia models.

## 3 Results

### 3.1 Population Genetic Structure

Thirty‐six haplotypes from 23 segregating sites were identified in the North American Arctic islands (NAAI) mitochondrial CR sequence (135 bp) and 67 haplotypes from 34 segregating sites were identified in the Barents Sea islands (BSI) mitochondrial sequence (204 bp). The West Greenland and extinct East Greenland sampling populations had the lowest gene diversities and allelic richness compared to other populations within the NAAI. Svalbard gene diversity and allelic richness were also relatively low, but the Sakha Republic had the lowest gene diversity; see Tables A5.1–A5.4 for genetic summary statistics of original sampling populations.

**FIGURE 1:**
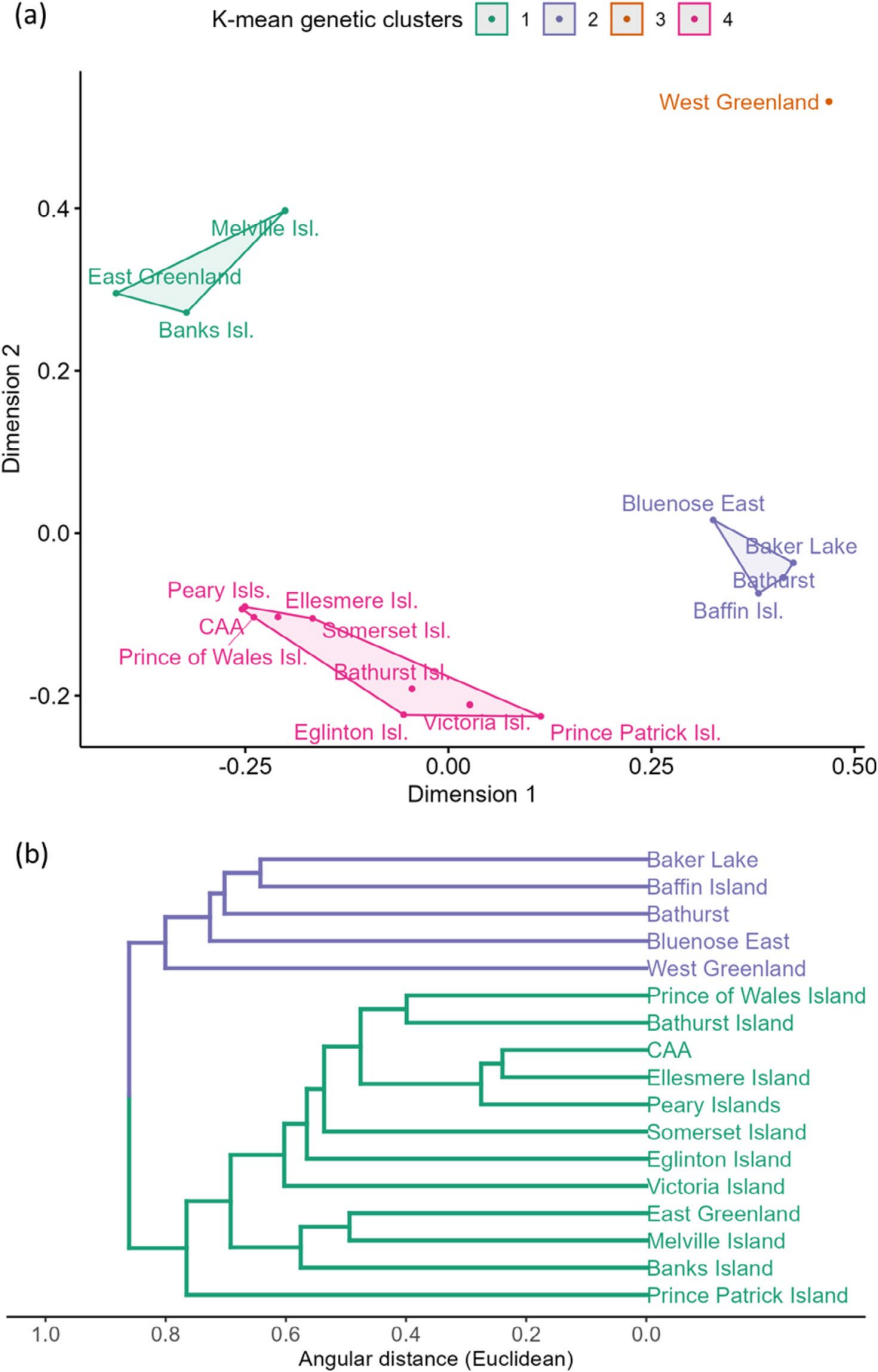
Population genetic clustering of *Rangifer tarandus* for the North American Arctic islands (NAAI) and neighbouring mainland populations. (a) Ordination plot of metric multidimensional scaling (mMDS) of original sampling populations. mMDS represents distances between populations with the lowest possible dimensional space; populations closer together are more similar than those further apart. Colours represent the optimal *K*‐means clusters (*K* = 3) identified using the average silhouette method on the mMDS coordinates (*n* = 317). (b) Dendrogram of original sampling populations based on a UPGMA hierarchical clustering method where populations that are genetically closer to each other cluster together. Colours represent the optimal *K*‐means clusters (*K* = 2) identified using the average silhouette method on the Euclidean distance matrix of mitochondrial sequence data (*n* = 317).

Hierarchical clustering using UPGMA based on pairwise Euclidean differences identified two main clusters in the NAAI, where the mainland and West Greenland populations clustered separately to the CAA island populations and East Greenland. The *K*‐means analysis partitioned individuals into two groups that corresponded to these two main clusters (Figure 1b). Principal coordinates analysis (PCoA) of pairwise genetic distances overlaid with *K*‐means clustering suggested four clusters (Figure 1a). The first principal coordinate accounted for 30.9% of the variation and separated the island and mainland populations, while the second accounted for 18.5% of the variation and separated an East Greenland, Banks Island, and Melville Island cluster from the rest of the CAA. These populations also shared low pairwise *F*_ST_ values, indicating that they were not genetically differentiated (Table A5.3). This pattern was likely affected by the presence of only one haplotype in the extinct East Greenland population, which was shared with Banks Island and Melville Island in addition to some other populations from the CAA. Unlike the UGPMA, the PCoA resolved West Greenland as a distinct cluster.

In the Barents Sea islands (BSI), the UPGMA identified two main clusters, with the western Russian populations forming a subgroup within the cluster containing the island populations. The *K*‐means analysis partitioned individuals into two groups that corresponded to these two main clusters (Figure A6.1). The PCoA *K*‐means clustering also revealed two main clusters (Figure A6.2). The first principal coordinate accounted for 32.5% of the variation and separated the island and mainland populations, while the second accounted for 14.7% of the variation and separated the island populations, with South Spitsbergen clustering with Novaya Zemlya and Franz Josef Land while Nordaustlandet formed its own outlier.

**FIGURE 2:**
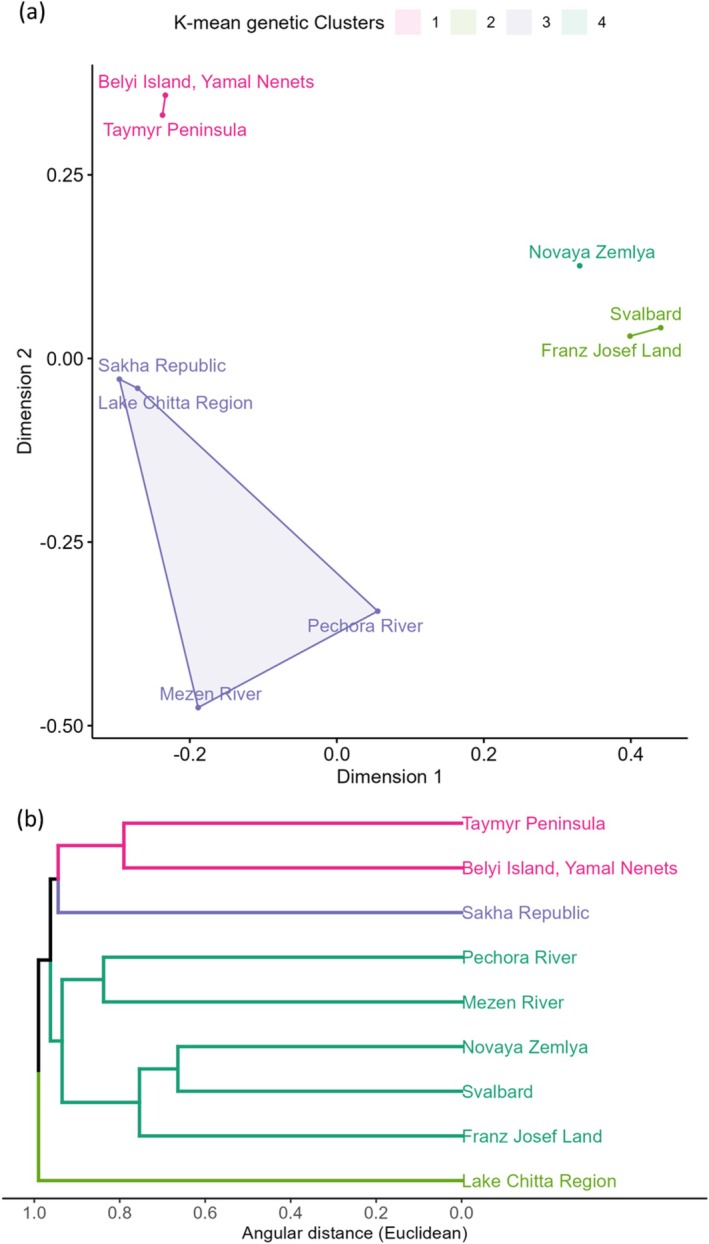
Population genetic clustering of *Rangifer tarandus* for the Barents Sea islands (BSI) and neighbouring mainland populations with Svalbard populations grouped. (a) Ordination plot of metric multidimensional scaling (mMDS) of sampling populations. mMDS represents distances between populations with the lowest possible dimensional space; populations closer together are more similar than those further apart. Colours represent the *K*‐means clusters (*K* = 4) identified using the average silhouette method on the mMDS coordinates (*n* = 335). (b) Dendrogram of sampling populations using a UPGMA hierarchical clustering method based on pairwise Euclidean distances, where populations that are genetically closer to each other cluster together. Colours indicate non‐hierarchical *K*‐means clusters (*K* = 4) identified using the average silhouette method (*n* = 335).

To better distinguish relationships between the mainland populations, the population clustering analyses were repeated with all Svalbard populations grouped together. All clustering approaches identified four populations, although the groupings differed between the methods, with the UPGMA *K*‐means separating Eastern Russian populations, with Sakha Republic clustering with northern populations (Figure 2b). The UPGMA *K*‐means also grouped western Russia with the island populations, while the first principal coordinate of the PCoA separated the island populations from the mainland (accounting for 21% of the genetic variation). The second principal coordinate separated the mainland populations into north, east, and west (17% of variation), although *K*‐means grouped east and west together (Figure 2a).

### 3.2 Comparing Demographic History and Postglacial Ice Sheet Retreat

#### 3.2.1 North American Arctic Islands

**FIGURE 3:**
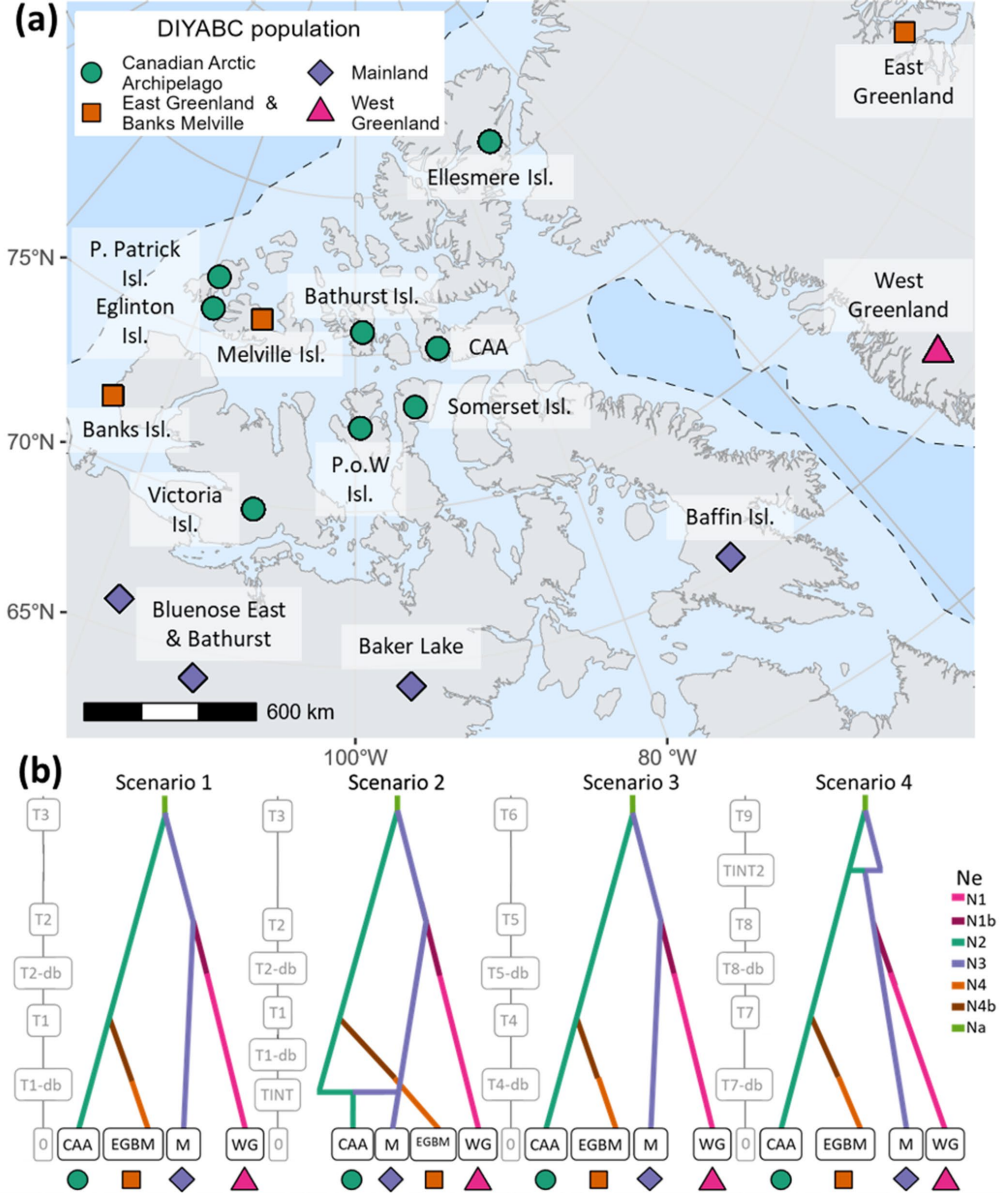
*Rangifer tarandus* in the North American Arctic islands (NAAI) system and final four evolutionary scenarios. Map of approximate geographic sampling locations (a), colour‐coded by the population groupings used for demographic analysis. Dotted line indicates the best estimate of LGM ice sheet extent. P. Patrick Isl. = Prince Patrick Island. P.o.W Isl. = Prince of Wales Island. Map projection North Pole Lambert azimuthal equal‐area Canada. (b) Evolutionary scenarios of the final model set tested with DIYABC RF. Scenario two was the preferred model (0.828 posterior probability). Ne is the haploid effective population size of each population, with population bottlenecks in East Greenland & Banks Melville (N4b) and West Greenland (N1b) after divergence from ancestral populations.

The final set of NAAI models contained four populations (Figure 3); see Tables A7.1 and A7.3 for genetic summary statistics for each population. Scenario two, which showed CAA and mainland populations diverging from an ancestral mainland population, and featured admixture between the CAA and mainland, had the highest posterior probability (0.828); see Table A4.3 for model error rates and Figure 5 for LDAs of observed versus simulated data for each scenario. The extinct East Greenland samples clustered in a group with the extant Banks and Melville Island herds. Whereas various model scenarios were tested with East Greenland as a separate population, in the preferred scenario these groups were combined into one population (East Greenland & Banks Melville). The sampling time of this population was set to zero generations as the East Greenland samples were collected in 1892 (Gravlund et al. 1998): less than 15 generations in the past, assuming a generation time of 7 years (Klütsch et al. 2017; Polfus et al. 2017). The best supported model suggested population divergence between the CAA and the mainland populations during the early Holocene at 10.7 ka (95% CI 7.4–13.7 ka), coinciding with the retreat of the Laurentide ice sheet and parts of the western CAA becoming ice free (Dalton et al. 2020).

The West Greenland–mainland population divergence was estimated at 4.9 ka (95% CI 1.5–10.1 ka), although the coasts of Baffin Island and West Greenland were deglaciated from ca. 8.5 ka (Dalton et al. 2020). Baffin Island was included in the mainland group in the final model set, as Baffin Island *Rangifer* clustered with Baker Lake in the mMDS and shared a low pairwise *F*_ST_ value with Baker Lake (Table A5.3), indicating the populations were not genetically differentiated. The best supported model also included an introgression (unidirectional admixture) event from the mainland population into the CAA population (2.1 ka, 95% CI 1.1–5.2 ka); see Table A8.1 for all parameter estimates.

#### 3.2.2 Barents Sea Islands

**FIGURE 4:**
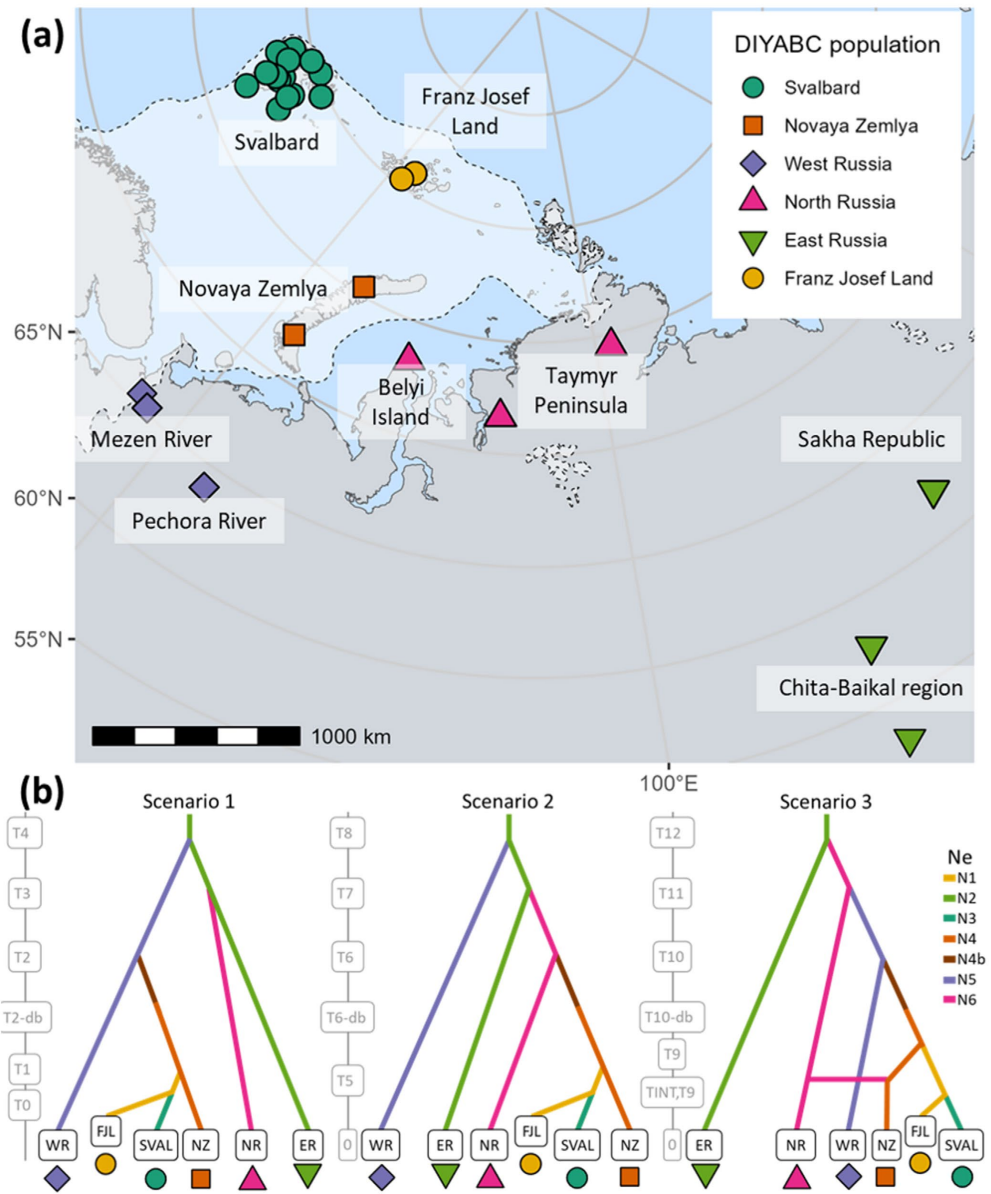
*Rangifer tarandus* in the Barents Sea islands (BSI) system and final three evolutionary scenarios. Map of approximate geographic sampling locations for *R. tarandus* (a), colour‐coded by population groupings used for demographic analysis. Dotted line indicates the best estimate of LGM ice sheet extent (Batchelor et al. 2019). Map projection North Pole Lambert azimuthal equal‐area Russia. (b) Evolutionary scenarios of the final model set tested with DIYABC RF. Scenario one was the preferred model (0.825 posterior probability). Ne refers to haploid effective population size of each population, with population bottleneck (N4b) in the Novaya Zemlya ancestral population after divergence from ancestral populations.

**FIGURE 5:**
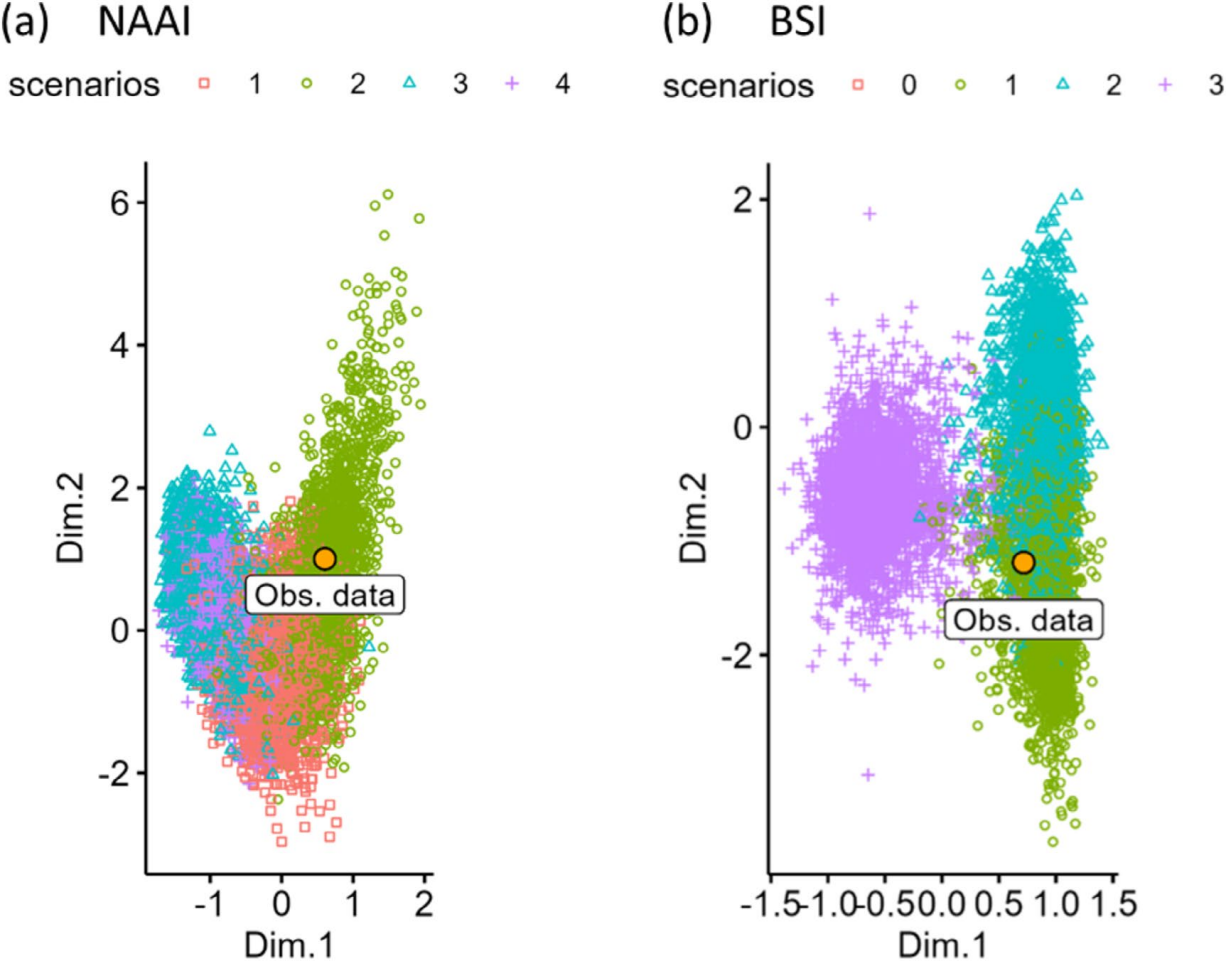
Projection of *Rangifer tarandus* simulated genetic datasets from the final DIYABC RF model set on the first two axes of a Linear Discriminant Analysis for (a) North American Arctic islands (NAAI) and (b) Barents Sea islands (BSI). Each scenario is plotted by colour, with the location of the observed genetic data set, situated within the cloud of points for the preferred scenario, is indicated by the yellow circle.

The final set of BSI scenarios contained six populations (Figure 4); see Tables A7.2 and A7.4 for genetic summary statistics. Scenario one had the highest posterior probability (0.83); see Table A4.3 for model error rates and Figure 5 for LDAs of observed versus simulated data for each scenario. Scenario 1 showed populations diverging from an ancestral East Russian population during the last glacial period (47 ka, 95% CI 21.2–92.9 ka; see Table A8.2 for all time parameter estimates). Although the southern island of Novaya Zemlya was likely ice free by 18 ka (Hughes et al. 2016), the Novaya Zemlya population did not diverge from mainland populations until 10.3 ka (95% CI 4.8–19.4 ka). Similarly, the Svalbard–Franz Josef Land divergence was estimated at 6 ka (95% CI 3.7–9.6 ka) and Franz Josef Land diverged from Novaya Zemlya at 7.8 ka (95% CI 4.3–10.8 ka), considerably after ice sheet retreat from Svalbard coasts at 12 ka and from Franz Joseph Land at 10 ka (Hughes et al. 2016). A Novaya Zemlya–North Russia admixture event was included in scenario three of the final model set because of lower genetic differentiation between Novaya Zemlya and the Taymyr Peninsula compared to Svalbard (Table A5.4), but this was not the best supported scenario in the RF model choice analysis.

### 3.3 Comparing Demographic History and Postglacial Sea Ice Changes

We compared the temporal correspondence of key events from the demographic history of North American Arctic islands (NAAI) and the Barents Sea islands (BSI) *Rangifer* to modelled and proxy‐based postglacial sea ice and ice sheet changes.

#### 3.3.1 North American Arctic Islands (NAAI)

**FIGURE 6:**
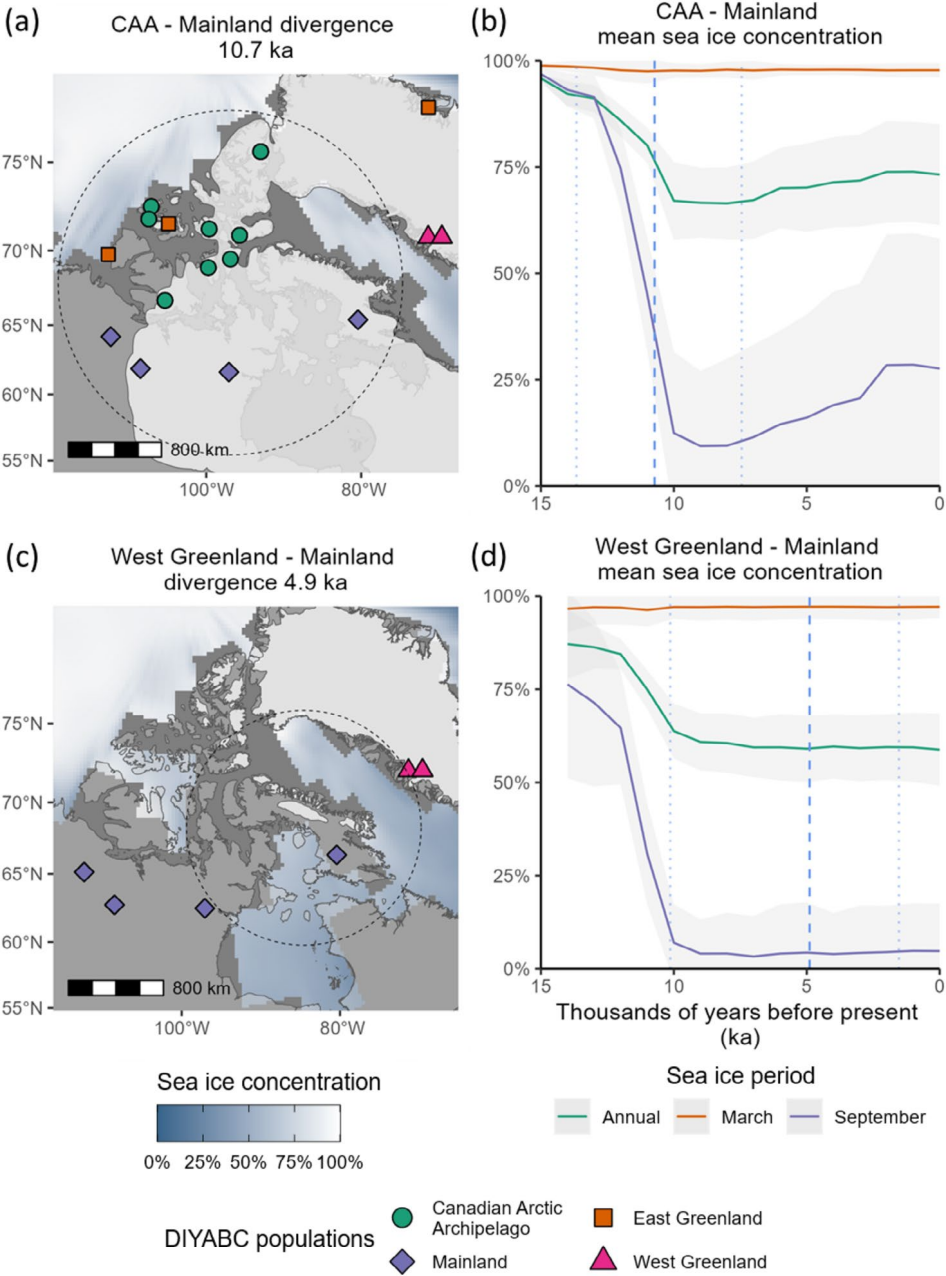
*Rangifer tarandus* population divergence events and modelled sea ice changes in the North American Arctic islands (NAAI). (a, c): Demographic events mapped with relevant present‐day populations, modern land extent (light grey), a snapshot of modelled mean sea ice concentration (blue—white) and land extent (dark grey) at 11 ka (a) and 5 ka (c), and reconstructed ice sheets at 10.9 ka (a) and 4.5 ka (c) – the closest time steps to the corresponding population events. Divergence time estimates are the median parameter estimates from the most plausible evolutionary model (scenario two). The geographic centroid between two populations (solid circle), within which 100 random samples of modelled sea ice concentration at different radii (dotted circle) were extracted and averaged (one radius shown for clarity). (b, d): Modelled sea ice concentration with associated standard deviations in 1 ka intervals. Demographic event times (dashed lines) with associated 95% credible intervals (dotted lines). Map projection North Pole Lambert azimuthal equal‐area Canada.

The CAA–mainland divergence occurred during or following a period of modelled annual and summer sea ice decline, although spring sea ice cover remained above 90% (Figure 6). Postglacial divergence of the island populations from the mainland occurred during large declines in modelled sea ice cover from 13 to 10 ka (Figure 6b). Sea ice cover therefore fell below the 70% minimum threshold for crossing (Mallory and Boyce 2018; Poole et al. 2010) between June and October (Figure A9.1).

Sedimentary core analyses suggest ice sheet retreat and a commencement of a marine environment within the channels of the CAA by ca. 10.8 ka (Pieńkowski et al. 2012, 2013). Our paleoclimate model data did not show modern land configuration until 6 ka, and was further limited in its spatial resolution, resulting in the absence of sea ice data for some CAA channels (Figure 6a,c). Proxy reconstructions show that the Nares Strait between East Greenland and Ellesmere Island was inundated before 9 ka, and the Barrow Strait in the CAA was inundated prior to 7.8 ka (Briner et al. 2016).

**FIGURE 7:**
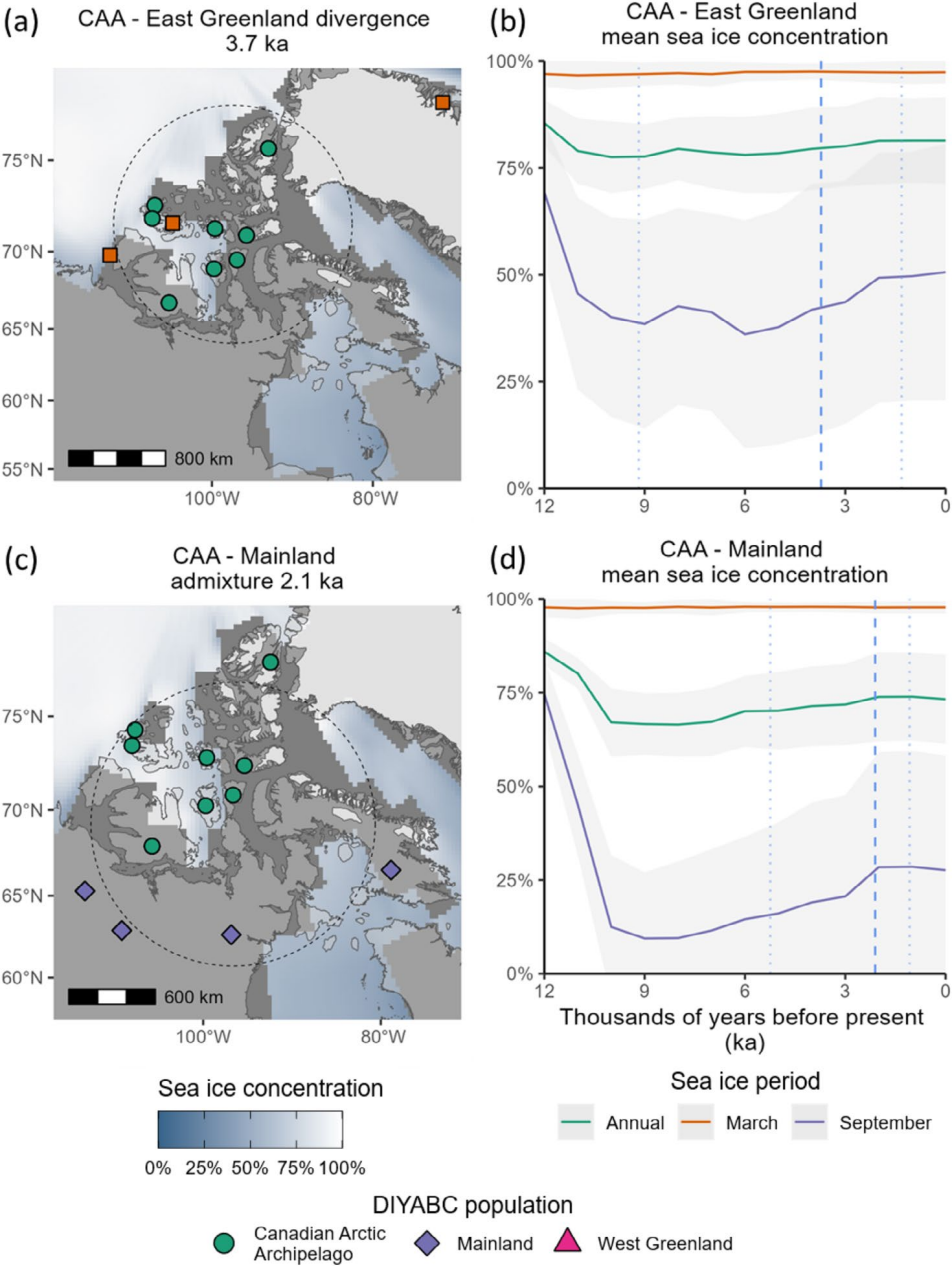
*Rangifer tarandus* population divergence and admixture events, and modelled sea ice changes in the North American Arctic islands (NAAI). (a, c): Demographic events mapped with relevant present‐day populations, modern land extent (light grey), a snapshot of modelled mean sea ice concentration (blue—white) and land extent (dark grey) at 4 ka (a) and 2 ka (c), and reconstructed ice sheets at 3.2 ka (a) and 2 ka (c) – the closest time steps to the corresponding population events. Divergence time estimates are the median parameter estimates from the most plausible evolutionary model (scenario two). The geographic centroid between two populations (solid circle), within which 100 random samples of modelled sea ice concentration at different radii (dotted circle) were extracted and averaged (one radius shown for clarity). (a) Divergence and (c) admixture time estimates mapped with relevant present‐day populations. (b, d): Modelled sea ice concentration with associated standard deviations in 1 ka time intervals. Timing of demographic events (dashed lines) with associated 95% credible intervals (dotted lines). Map projection North Pole Lambert azimuthal equal‐area Canada.

The West Greenland–mainland divergence occurred during a period of stable but relatively low ice cover with ice‐free autumns, but with March sea ice concentrations close to 100% (Figure 7b). Proxy‐based reconstructions suggest that spring sea ice cover was present throughout the Holocene between Baffin Island and West Greenland (Saini et al. 2022), and document an increase in sea ice cover from ca. 5.5 ka in northern Baffin Bay, and a significant cooling pulse in eastern Baffin Bay ca. 4.5 ka (Briner et al. 2016), just prior to the modelled West Greenland Rangifer divergence time. However, widespread neoglacial cooling appears to commence ca 3 ka (Saini et al. 2022).

The East Greenland & Banks Melville–CAA divergence at 3.7 ka (95% CI 1.3–9.1 ka) also occurred during the onset of the neoglacial, during a period of worsening climate. Proxy‐based reconstructions suggest reduced sea ice cover from 9 to 6 ka in the Nares Strait that separates Ellesmere Island from Greenland, followed by increased sea ice cover from 6 ka (Briner et al. 2016; Jennings et al. 2011). The CAA‐mainland admixture event at 2.1 ka (95% CI 1.1–5.2 ka) occurred during the neoglacial and was coeval with a small increase in modelled ice cover in the summer and early autumn months (Figure 7d and Figure A9.2).

#### 3.3.2 Barents Sea Islands

Modelled sea ice shows no clear change in sea ice extent during the Novaya Zemlya–West Russia divergence, with some months experiencing an increase in sea ice and other months stable or decreasing (Figure A9.3). Ice sheet reconstructions suggest ice sheets had already retreated from this potential crossing region by ca. 16 ka (Hughes et al. 2016). Proxy‐based records of relative sea level suggest that relative sea level on the South Island of Novaya Zemlya was ~35–50 m higher than present from ca. 18–3 ka, indicating that a land bridge may not have been present (Baranskaya et al. 2018).

**FIGURE 8:**
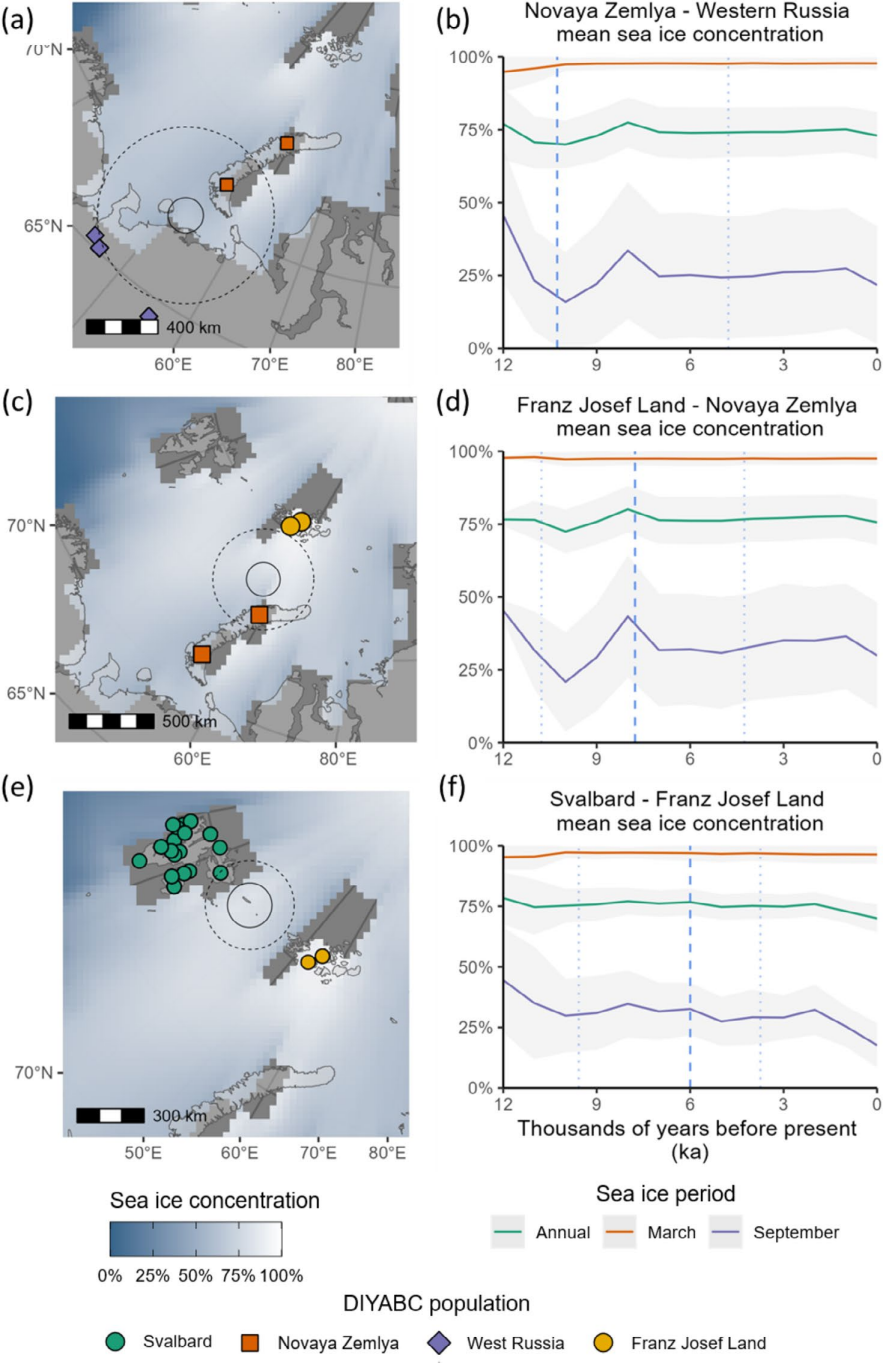
*Rangifer tarandus* population divergence and sea ice changes in the Barents Sea islands system (BSI). (a, c, e): Demographic events mapped with relevant present‐day populations, modern land extent (light grey), a snapshot of modelled mean sea ice concentration (blue—white) and land extent (dark grey) at 10 ka (a) 8 ka (c) and 6 ka (e). Divergence time estimates are the median parameter estimates from the most plausible evolutionary model (scenario three). The geographic centroid between two populations (solid circle), within which 100 random samples of modelled sea ice concentration at different radii (dotted circle) were extracted and averaged (one radius shown for clarity). (b, d, f): Modelled sea ice concentration with associated standard deviations in 1 ka intervals. Estimated population divergence time (dashed lines) with associated 95% credible intervals (dotted lines). Map projection North Pole Lambert azimuthal equal‐area Russia.

Our modelled Franz Josef Land–Novaya Zemlya population divergence occurred just after an increase in annual sea cover to over 75% (Figure 8), although sea ice concentration was below 70% from June to October during this period (Figure A9.3). The Svalbard–Franz Josef Land population divergence was also associated with annual modelled sea ice cover of ca. 75% (Figure 8).

Proxy reconstructions support modelled spring sea ice cover persisting in the northern Barents Sea (Pieńkowski et al. 2021), with spring and summer sea ice present between Franz Josef Land and Nordaustlandet (north‐eastern Svalbard) throughout the Holocene (Berben et al. 2017). However, the IP_25_ proxy‐based estimates of spring sea ice concentrations are consistently lower than our model‐derived estimates, falling below 50% from ca. 12 to 8 ka during the main deglaciation of Svalbard, before increasing to ca. 60% by ca. 6 ka (Berben et al. 2017; Pieńkowski et al. 2021), close to our modelled divergence time of Svalbard Rangifer.

## 4 Discussion

Our results show a complex Late Quaternary history for Arctic island *Rangifer*, with Holocene dispersal from continental glacial refugia to deglaciated islands followed by a varying degree of secondary contact with mainland populations. Dispersal likely occurred in a stepwise fashion for both regions, as evidenced by the closer genetic relationships in geographically closer populations. The best‐supported models in the NAAI included genetic admixture in the late Holocene (2.1 ka) between the Canadian Arctic Archipelago and the mainland populations during a cooling period in the Arctic (Briner et al. 2016). The divergence time of the NAAI island populations from the mainland coincided with ice sheet retreat from western islands, but the West Greenland divergence and the BSI Novaya Zemlya and Svalbard population divergences occurred millennia after deglaciated land was available, implicating other factors such as sea ice conditions and drift in determining their postglacial dispersal.

### 4.1 High Arctic Refugium or Postglacial Dispersal?

Our results do not support High Arctic glacial *Rangifer* refugia—i.e. the persistence of small populations in isolated ice‐free regions—for island *Rangifer* in either region (if these existed, they did not contribute to the current populations). Instead, the ancestral populations of contemporary NAAI *Rangifer* probably spent the last glacial period in a common Beringian location with mainland populations.

Our results contrast with coalescent‐based modelling studies that proposed the existence of one or more High Arctic glacial refugia in the CAA (Eger et al. 2009; Klütsch et al. 2017). The presence of a High Arctic refugium during the Last Glacial Maximum (LGM, ca. 21 ka BP) that would be large enough to support *Rangifer* populations has largely been discarded based on updated compilations of ice sheet chronologies, which show complete ice coverage of the hypothesised refugium on Banks Island (Dalton et al. 2022). Regardless, ice‐free areas in the CAA may not have been capable of sustaining a *Rangifer* population if conditions were too dry and cold for sufficient forage biomass, as has been suggested for Greenland (Gravlund et al. 1998).

Our results from the BSI agree with other genetic work that has suggested Svalbard was colonised from what is currently Russia via Novaya Zemlya during the postglacial period (Dussex et al. 2023; Gravlund et al. 1998; Hold et al. 2024; Kvie et al. 2016). Our best‐supported model suggested that the ancestral BSI population diverged from the Eurasian mainland *Rangifer* 10.3 ka (95% CI 4.8–19.4 ka), which contrasts with phylogenetic analysis of whole mitogenomes suggesting that this lineage had already diverged from the main Euro‐Beringean lineage ca. 14.5–25.7 ka (Hold et al. 2024).

We did not find evidence for a common Beringean glacial location in eastern Siberia, as was proposed previously (Flagstad and Røed 2003; Kvie et al. 2016), and found instead that northern Eurasian populations may have been isolated in separate locations, with divergences between west and east in the glacial (47 ka, 95% CI 21.2–92.9 ka) and between north and east during the LGM (24.9 ka 95% 11.2–46.4 ka). We cannot rule out a scenario in which Eurasian populations were in a common glacial refugium, with *Rangifer* from Fennoscandia coming into postglacial contact with West Russian populations, contributing to their genetic distinctiveness (Baranova et al. 2012; Røed 2005). Therefore, the genetic relationships of the Russian *Rangifer* should be examined further with whole genomic markers to resolve the question of multiple glacial locations.

### 4.2 Holocene Divergence Times: Comparison With Subfossil Record and Molecular Studies

Divergence times in our BSI models broadly align with existing subfossil and molecular evidence of postglacial island colonisation by *Rangifer*. Previous published estimates of *Rangifer* arrival on Svalbard ranged from 6.7 to 5 ka (van der Knaap 1989), even though warmer climate conditions in the early to mid‐Holocene (9–6 ka) would have been able to sustain *Rangifer* populations in Svalbard (Forman et al. 2000). Our model suggests the Svalbard population split from the Franz Josef Land population at 6 ka (95% CI 3.7–9.6). Recent coalescent modelling of genomes suggested a Svalbard–Novaya Zemlya population divergence at 6.2 ka (5th–95th percentiles: 3.5–11.9 ka) (Dussex et al. 2023), while a phylogenetic analysis of modern and ancient mitogenomes (Hold et al. 2024) suggested that the first divergence among Svalbard individuals occurred ca. 5.9 ka (95% Height Posterior Density of 4.4–7.6 ka). These timings are similar to our median divergence date, although we used different methods and captured less genetic variation with our single CR marker than other genomic studies (Dussex et al. 2023; Hold et al. 2024).

Our best supported model suggests a potential colonisation of Franz Josef Land as early as 7.8 ka (95% CI 4.3–10.8 ka). Franz Josef Land has been proposed as a stepping stone for *Rangifer* colonising Svalbard due to Holocene subfossils dated to 6.4 ka (Forman et al. 2000) and ancient DNA mitochondrial haplotypes in Franz Josef Land, which are closely related to contemporary and ancient Svalbard haplotypes (Hold et al. 2024; Kvie et al. 2016). Hold et al. (2024) suggested that the Svalbard population had already diverged from the Franz Joseph Land/Novaya Zemlya lineage by 8.8 ka (95% Height Posterior Density of 6.5–11.3 ka), indicating a potentially earlier colonisation of Svalbard and Franz Josef Land than our model suggests, although it is within the range of uncertainty of our time estimates.

For the NAAI, our modelled Mainland‐West Greenland divergence time was 4.9 ka (95% CI 1.5–10.1 ka), while the first subfossil occurrences of large‐bodied *Rangifer* in West Greenland are documented ca. 4 ka (Bennike 1997). However, the earliest subfossil occurrence of small‐bodied reindeer *Rangifer* in southwest Greenland occurs from 5.8 ± 0.08 cal ka BP (Bennike 1997), which was likely related to the Peary Caribou of the CAA and East Greenland and died out after the large‐bodied reindeer arrived (Seersholm et al. 2022).

Our model supports a divergence between the population that includes Banks/Melville Islands and the extinct East Greenland individuals with other CAA populations at 3.7 ka (95% CI 1.3–9.2 ka), which is substantially after the first Holocene appearance of subfossils in North Greenland at 7.98 ± 0.115 cal ka BP (Knuth 1984) and the first appearance of *Rangifer* in East Greenland 6.2 ± 0.07 cal ka BP (Håkansson 1978). This divergence could indicate a halt in genetic connectivity between populations in the CAA and those in Eastern Greenland as the climate conditions deteriorated in the Neo‐Glacial, through an environmental filter in northernmost CAA and northern Greenland, effectively isolating Eastern Greenland *Rangifer*. Interestingly, a recent mitogenome‐based phylogeny places a Bathurst Island individual as sister group to East Greenland individuals, with a divergence time ca. 4 ka. The authors suggest this grouping could be a result of local adaptation (Hold et al. 2024). Alternatively, this discrepancy in divergence estimates may be because our analysis did not resolve the extinct East Greenland individuals (*N* = 3) as a separate population from Banks/Melville Islands, and so the divergence date likely reflects a later split between Banks/Melville Islands and the CAA, rather than the initial colonisation.

### 4.3 Contrasting Effects of Sea Ice on NAAI Postglacial History

On the basis of sea ice‐mediated genetic connectivity (Jenkins et al. 2016), we expected population divergences in the NAAI to be associated with reduced sea ice concentration, reflecting reduced inter‐population connectivity rather than island colonisation events. Accordingly, we found that the CAA population postglacial divergence occurred during a period of strong sea ice decline. *Rangifer* in the CAA currently require estimated sea ice cover percentages > 70% to make seasonal migrations (Bowler et al. 2025; Mallory and Boyce 2019; Poole et al. 2010). These levels presently occur from April to June and from September to November (Jenkins et al. 2016). Modelled sea ice reductions coinciding with divergences were strongest between July and October, with sea ice cover only remaining above 75% from October/November through to June. A reduction in the length of the migration season could have reduced spatiotemporal overlap between groups at autumnal rutting grounds (Dauphiné and McClure 1974), leading to reproductive isolation and genetic divergence at a time when more land in the CAA was available due to ice sheet decline.

However, the East Greenland & Banks Melville—CAA divergence was associated with increasing modelled sea ice that coincides with the cooling associated with the Neoglaciation (Briner et al. 2016), suggesting that worsening climatic conditions may have isolated populations. The increase in proxy‐based sea ice cover—although not modelled sea ice concentration—coincided with our modelled West Greenland divergence, which agrees with our expectation that *Rangifer* connectivity between distant land masses would be associated with periods of enhanced sea ice cover (see Section 4.4).

The best supported NAAI scenario included dispersal and gene flow from the mainland to the CAA at 2.1 ka (95% CI 1.1–5.2 ka). Proxy reconstructions suggest increased sea ice cover in eastern CAA commencing prior to the admixture event (Briner et al. 2016), although we found no substantial increase in modelled sea ice concentration, which would be expected to promote dispersal and admixture. This event could have taken the form of individuals from the mainland population contributing to the gene pool of herds such as the Dolphin‐Union herd on Victoria Island, which is recognised as an intermediate subspecies *R. t. groenlandicus* × *pearyi* (COSEWIC 2011). Analysis of microsatellite data has found evidence of historical gene flow between the Dolphin‐Union and mainland herds (McFarlane et al. 2016). Alternatively, admixture may have occurred on Baffin Island: the placement of Baffin Island within the CAA or the mainland genetic clusters varied depending on the clustering method used. Victoria Island and Baffin Island are only separated from the mainland by narrow, ice‐rich channels that cannot be resolved at the resolution of our paleoclimate model. For this reason, it is safe to assume that they have generally been well connected to the mainland throughout most of the Holocene.

### 4.4 Sea Ice‐Mediated Long‐Distance Dispersal of *Rangifer*

In the BSI, we expected high or increased sea ice cover to be associated with population divergence, representing long‐distance dispersal and island colonisation. The divergence of Franz Josef Land from Novaya Zemlya at 7.8 ka (95% CI 4.3–10.8 ka) immediately followed a period of moderate increase in summer and autumn sea ice cover in the potential crossing region. An increase in spring sea ice concentration at that time is also inferred from proxy data (Berben et al. 2017; Pieńkowski et al. 2021). However, the Svalbard–Franz Josef Land divergence at 6 ka (95% CI 3.7–9.6) and Novaya Zemlya–Mainland divergences at 10.3 ka (95% CI 4.8–19.4 ka) were not associated with a period of increased modelled sea ice cover. The latter might be explained by the short distance separating Novaya Zemlya from the Eurasian mainland (currently 56 km), which was likely narrower with possible emerged land in between during the early Holocene, before reaching approximately its current dimensions by 7 ka (Forman et al. 2004).

An enhanced Transpolar Drift current with greater ice drift velocities from ca. 7 ka (Hole and Macias‐Fauria 2017) may have favoured *Rangifer* dispersal westwards from Franz Joseph Land to Svalbard. Furthermore, the strong westerly drift may have prevented *Rangifer* from returning to Franz Joseph Land, which, along with worsening climate conditions in the Neoglacial, may explain the absence of *Rangifer* subfossils after 1.3 ka (Forman et al. 2000).

The large time interval between the Svalbard deglaciation (12–10 ka) and estimated Svalbard population divergence at 6 ka (95% CI 3.7–9.6 ka) indicates that the founders of the contemporary Svalbard population may not have been able to disperse to Svalbard earlier. This lag may be due to unfavourable sea ice conditions; proxy‐based sea ice reconstructions show low spring sea ice cover in the northern Barents Sea between 12 and 8 ka (Berben et al. 2017; Pieńkowski et al. 2021). This lag could also be a result of earlier unsuccessful colonisations that did not contribute to the contemporary gene pool.

In the NAAI, the West Greenland population divergence similarly appears to represent a scenario of long‐distance dispersal. It is unlikely that western Greenland was reached from the CAA via the Nares Strait and North Greenland, as ice sheets extended to the sea in this region throughout the Holocene (Dalton et al. 2020). The genetic data and best supported evolutionary scenario suggest a crossing from Baffin Island over the Davies Strait: ~300 km across at its narrowest point. Paleoenvironmental proxies suggest an increase in sea ice cover around the modelled divergence date, but no associated increases in modelled sea ice cover were evident. Favourable west–east ocean currents and sea ice drift direction may have also been important, although unfavourable east–west currents are present today (Wu et al. 2013).

Long distance dispersal of *Rangifer* to islands on sea ice resulting in the establishment of viable populations is likely a rare and random phenomenon, and therefore difficult to associate with large‐scale sea ice trends (Gillespie et al. 2012; Jordano 2017), as was done for likely dispersal routes to Arctic islands coinciding with Holocene first occurrences of plant species (Alsos et al. 2016). *Rangifer* regularly makes short sea ice crossings in the Canadian Arctic, within Svalbard, and in the New Siberian islands (Jenkins et al. 2016; Mizin et al. 2018; Peeters et al. 2019; Poole et al. 2010) but are less well‐adapted for long‐duration, long‐distance crossings. However, dispersal over longer distances in the CAA (ca. 300–400 km) is occasionally recorded (Miller et al. 2005).

There are also historical accounts of the sporadic movement of individual *Rangifer* from Novaya Zemlya to Svalbard. A large male was shot in Sassendalen, Svalbard, in October 1911 and its antler was found to be tagged in a way that was associated with Nenets *Rangifer* herders of Novaya Zemlya (Hoel 1916; Lønø 1959). A crossing of over 700 km as the crow flies between Novaya Zemlya and Svalbard was constrained between antler regrowth (approximately May) and sea ice annual minimum extent (August/September) in what was reportedly a year with particularly extensive ice coverage in Svalbard (Lønø 1959). These anecdotes support the hypothesis that long‐distance migrations over ice are possible, if rare.

### 4.5 Limitations

Mitochondrial DNA has been commonly used to study the demographic history of natural populations because of its relatively rapid rate of mutation, which is useful for inferring events on Pleistocene timescales (Norman et al. 2014). However, this high mutation rate can lead to recurrent mutations (homoplasy), which together with substantial among‐site rate heterogeneity can obscure genealogical relationships and affect divergence times (citations). To address this, we chose the HKY substitution model (Hasegawa et al. 1985) with extensions that account for multiple substitutions at a single site, invariable sites, and gamma‐distributed mutation rate variation among sites (Collin et al. 2021).

The mitochondrial genome can also be subject to selective forces (Balloux 2010) which lead to departures from the neutral equilibrium expectations of coalescent models, which may have influenced our results. As mentioned earlier, the clustering of Banks Island, Melville Island, and East Greenland populations in one group may be due to sequence similarity due to common selective pressures (Hold et al. 2024) rather than recent divergence, given their geographical distance today. Additional work using putatively neutral nuclear markers could help resolve the evolutionary relationships between these populations.

Our use of single‐locus mitochondrial control region also reflects only the maternal lineage and a single genealogical pathway, potentially leading to incomplete, biased, or low‐resolution reconstructions of population history (Balloux 2010). However, comparing our results with nuclear (Dussex et al. 2023) and mitochondrial (Hold et al. 2024) whole genome studies showed similarities in genetic clustering and the timing of the colonisation of Svalbard. Our control region sequences seem to infer an overall similar pattern of demographic history to multilocus markers, including nuclear markers, which are more likely to be selectively neutral.

This study used mitochondrial sequences from published literature, where individuals were associated with a population, herd, or locality based on sampling location, Using pre‐defined populations (e.g., based on geographical proximity) for demographic modelling can be problematic because genetic variation in *Rangifer tarandus* often does not reflect present‐day subspecies designations, geographical populations, or ecotypes (Polfus et al. 2017; Serrouya et al. 2012; Weckworth et al. 2012). Arbitrary assignment to populations can lead to more genetic variation and structure within populations than between populations, making genetic data simulation using coalescent modelling challenging. However, we did not reassign populations based on individual genetic clustering as could have led to biologically unrealistic populations due to genetic similarities between geographically distant populations resulting from the short sequence length of the mitochondrial CR locus.

Uncertainty in demographic events could be improved by using the richer information content provided by whole mitogenomes, which although now available for the Barents Sea region, are sparse in the North American Arctic islands (Hold et al. 2024). Integrating nuclear genomic data from the BSI (Dussex et al. 2023) and NAAI (Taylor et al. 2024) would also provide a more complete picture of demographic history than mitochondrial data alone can provide (Balloux 2010) and improve the robustness of divergence time estimates which are dependent on the type of marker used (Hold et al. 2024; Polfus et al. 2017). Including ancient DNA in demographic analyses could further improve the accuracy of demographic parameters, particularly in Svalbard, as historical overharvest led to bottlenecks and haplotype loss (Kellner et al. 2024). Ancient DNA haplotypes that are not present in the modern population could lead to alternative narratives.

### 4.6 Conclusions

This study sheds light on the glacial and postglacial history of Arctic island *Rangifer* and suggests sea ice played a role in shaping present population genetic patterns. Geographical, environmental, and climatic differences between the North American Arctic islands and the Barents Sea islands contributed to different postglacial *Rangifer* dynamics. Unlike the isolated archipelagos of the BSI, reduced genetic structure and modelled admixture events in the NAAI suggest that populations were connected to some degree throughout the Holocene via dispersal across narrow, ice‐rich ocean passages (Mallory and Boyce 2018). Linked to this, we found evidence of low sea ice cover coinciding with genetic divergence between populations in the CAA, with late spring and autumn sea ice cover likely important in promoting connectivity. In the BSI, and despite no consistent modelled sea ice signal preceding *Rangifer* population island colonisation, proxy‐based evidence suggests that increases in spring sea ice cover and favourable ocean current direction and speed enabled dispersal to Franz Joseph Land and Svalbard.

Postglacial and early Holocene divergences in the NAAI appear to follow retreating ice sheets. Yet both the West Greenland and Barents Sea island populations, which required the longest on‐sea migration, diverged long after ice‐free areas in their present‐day ranges were available, suggesting either (i) dispersal limitation due to unfavourable sea ice conditions, (ii) time lags resulting from the low probability of rare long‐distance dispersal events, (iii) uncertainty in the timing of demographic events resulting from the resolution of the genetic data, or (iv) a combination of some of the above.

The difference in historic population connectivity in each region has implications for the persistence of *Rangifer* under future climate change. Seasonal, land‐fast ice cover is predicted to persist to some extent in the CAA (Newton et al. 2021), enabling some connectivity and potentially some movement of individuals from the southern CAA and mainland Canada (Mallory and Boyce 2018). The Svalbard *Rangifer* are much more geographically isolated than their counterparts in the CAA, in terms of both geographic distance from the mainland and sea ice connectivity. The maximum spring sea ice extent in the Barents Sea is projected to move north of Svalbard under ongoing climate change (Dörr et al. 2021; Shu et al. 2021): Svalbard will thus not be connected by sea ice to any land masses in the future, however ephemerally. Therefore, the realised dispersal of *Rangifer* may vary regionally and depend upon the progressively diminished connectivity provided by sea ice, especially over larger distances.

## Author Contributions

**Maria Dance:** conceptualization (lead), data curation (lead), formal analysis (lead), funding acquisition (equal), methodology (lead), software (lead), visualization (lead), writing – original draft (lead), writing – review and editing (lead). **Erin E. Saupe:** conceptualization (supporting), data curation (supporting), methodology (supporting), software (supporting), supervision (equal), writing – review and editing (supporting). **Alex Farnsworth:** data curation (supporting), writing – review and editing (supporting). **Paul J. Valdes:** data curation (supporting). **Marc Macias‐Fauria:** conceptualization (supporting), funding acquisition (equal), methodology (supporting), supervision (equal), writing – review and editing (supporting).

## Funding

This work was supported by Natural Environment Research Council, 1929306, NERC NE/L011859/1.

## Conflicts of Interest

The authors declare no conflicts of interest.

## Supporting information


**Appendix S1:** ece373125‐sup‐0001‐Appendices.docx.

## Data Availability

The data and code that support the findings of this study are available in Dryad at https://doi.org/10.5061/dryad.j9kd51cjf. Genetic data were derived from datasets published in NCBI Genbank (DOIs in Appendix S1). Accession numbers are in the online Supporting Information—S1. Paleoclimate model data can be accessed from the repository at www.bridge.bris.ac.uk/resources/simulations.
