# Supplementary material for: Retracing the Response of Rangifer to Postglacial Climate Change in Arctic Islands
Source: Ecol Evol. 2026 Mar 19;16(3):e73125. doi: 10.1002/ece3.73125 (PMC13093289; doi:10.1002/ece3.73125)
Supplement: Supplementary file 1 — Appendix S1: ece373125‐sup‐0001‐Appendices.docx. [file ECE3-16-e73125-s001.docx]

Appendices

Contents

[Appendix 1: Datasets 48](#_Toc210127702)

[Appendix 2: Evolutionary scenario development and model testing 49](#_Toc210127703)

[Appendix 3: Paleoclimate model specifications 64](#_Toc210127704)

[Appendix 4: DIYABC RF model choice output for final model sets 65](#_Toc210127705)

[Appendix A5: Genetic summary statistics of sampling populations 66](#_Toc210127706)

[Appendix 6: Population genetic clustering results 70](#_Toc210127707)

[Appendix 7: Genetic summary statistics of DIYABC model populations 71](#_Toc210127708)

[Appendix 8: Parameter estimates from final DIYABC RF models 73](#_Toc210127709)

[Appendix 9: Reindeer population events and modelled sea ice change 75](#_Toc210127710)

[Appendix References 76](#_Toc210127711)

# Appendix 1: Datasets

**Table A1.1: Details of published genetic data (mitochondrial control region) of Rangifer tarandus used in the analysis**.

| **Publication** | **Publication title** | **Publication DOI** | **Number of populations** | **Number of individuals** | **Spatial coverage** |
| --- | --- | --- | --- | --- | --- |
| Gravlund et al., 1998 | Polyphyletic Origin of the Small-Bodied, High-Arctic Subspecies of Tundra Reindeer (Rangifer tarandus) | 10.1006/mpev.1998.0525 | 8 | 98 | Circumpolar |
| Flagstad & Røed, 2003 | Refugial Origins of Reindeer (Rangifer tarandus L.) Inferred from Mitochondrial Dna Sequences | 10.1111/j.0014-3820.2003.tb01557.x | 7 | 89 | Circumpolar |
| Røed et al., 2008 | Genetic analyses reveal independent domestication origins of Eurasian reindeer | 10.1098/rspb.2008.0332 | 4 | 125 | Palearctic |
| Eger et al., 2009 | Genetic diversity and history of Peary caribou (Rangifer tarandus) in North America | https://www.academia.edu/  44005159/  PROCEEDINGS_FROM_THE_  CARIBOU_GENETICS_AND_  RELATIONSHIPS_WORKSHOP | 9 | 90 | American Arctic |
| Petersen et al., 2010 | Bottlenecks, isolation, and life at the northern range limit: Peary caribou on Ellesmere Island, Canada | 10.1644/09-MAMM-A-231.1 | 1 | 121 | CAA |
| Kvie et al., 2016 | Colonizing the High Arctic: Mitochondrial DNA Reveals Common Origin of Eurasian Archipelagic Reindeer (Rangifer tarandus) | 10.1371/journal.pone.0165237 | 6 | 92 | Barents region / Western Siberia |
| Røed et al., 2020 | Temporal and structural genetic variation in reindeer (Rangifer tarandus) associated with the pastoral transition in northwest Siberia | 10.1002/ece3.6314 | 1 | 36 | Western Siberia |
| Hold et al., 2024* | Ancient reindeer mitogenomes reveal island-hopping colonisation of the Arctic archipelagos | 10.1038/s41598-024-54296-2 | 26 regions, 38 locations. | 174 | Canada, Russia, Greenland, Svalbard, Franz Josef Land, Novaya Zemlya |

* Includes published sequences from: Burnett et al., 2023; Dussex et al., 2023; Kellner et al., 2024; Taylor et al., 2020

# Appendix 2: Evolutionary scenario development and model testing

**Table A2.1 DIYABC RF model specification common to all scenarios of Rangifer tarandus demographic history.**

| **Priors** | **Characteristics** | **Source** |
| --- | --- | --- |
| Contemporary population size (Haploid effective population size (Ne)) | Uniform distributions centred around census population sizes and estimates | (BQCMB, 2014; Cuyler, 2010, p. 20; Davison, 2016; Department of Environment, Government of Nunavut, 2013; Gunn, 2016; Kaluskar et al., 2020; Klütsch et al., 2017; Le Moullec et al., 2019; Mizin et al., 2018; Pedersen et al., 2019; Poole et al., 2010; Wekʼèezhìi Renewable Resources Board, 2023) |
| Admixture rate r_a_ (how the lineages of admixed population are distributed among the parental population) | Uniform prior distribution bounded between 0.1 and 0.75 (NAAI) and 0.01 and 0.5 (BSI) | Evidence of admixture between Victoria Island and mainland (McFarlane et al., 2016) and guidelines in DIYABC RF manual (Collin et al., 2021) |
| Divergence/admixture time | Uniform distributions of plausible broad ranges:  (i) Holocene - present day (x to x generations/years)  (ii) Postglacial from LGM 24,000 years BP - present day  (iii) Last glacial period from 100,000 years BP - present day | NA |
| Bottleneck duration | 100-1000 years (14-140 generations) after the relevant divergence time | NA |
| Bottleneck size (Haploid population size (Ne) during bottleneck) | 5-500 | NA |
| DNA mutational model | HKY nucleotide substitution model with gamma-distributed rate heterogeneity (unless otherwise specified) | Previous studies of reindeer mitochondrial markers (Eger et al., 2009; Flagstad & Røed, 2003; Kuhn et al., 2010; Letts et al., 2012; Røed et al., 2020) |
| DNA mean mutation rate | 1x10^-6^ - 1x10^-8^ per site per generation (unless otherwise specified) | (Eger et al., 2009; Flagstad & Røed, 2003; Kvie et al., 2016; Yannic et al., 2014) |

**Table A2.2 Evolutionary scenarios for Rangifer tarandus tested with DIYABC RF for the North American Arctic islands (NAAI) region.** Populations: CAA = Canadian Arctic Archipelago, EGBM = East Greenland & Banks Melville, BAFF = Baffin Island, M = Mainland, WG = West Greenland, VIC = Victoria Island (Dolphin Union herd). Model sets tested against each other using Random Forest model classification with DIYABC RF when the simulated datasets from the training set and from the observed dataset overlapped on the first two axes of a principal component analysis (PCA), as recommended in the DIYABC RF manual (Prior scenario checks; Collin et al., 2021). Model choice refers to the overlap between observed and simulated data for each scenario on linear discriminant analysis (LDA) axes. Model posterior error corresponds to prediction quality exactly at the position of the observed dataset, with lower values indicating lower error rates and therefore higher prediction quality. Posterior probability reflects the most plausible, or “best model” within the tested model set.

| **Model set** | **Population groups** | **Scenario number** | **Key events** | **Source** | **Details** | **Prior scenario checks (PCA)** | **Model choice (LDA)** | **Model posterior error** | **Posterior probability** |
| --- | --- | --- | --- | --- | --- | --- | --- | --- | --- |
| NAAI24 (10,000 datasets) | Pop 1: CAA (including VIC)  Pop 2: EGBM  Pop 3: M (including BAFF)  Pop 4: WG | 24.1 | Holocene divergence | Gravlund et al .,1998; Flagstad et al., 2003 |  | Scenario 1 and 2 closest to observed data. Scenarios 3 and 4 not distinct. | Observed data sits in between simulated data from scenario 1 and scenario 2 | 0.20 | 0.712 |
|  |  | 24.2 | Holocene divergence with CAA-M admixture | McFarlane et al., 2016 | Gene flow from CAA into mainland |  |  | 0.094 |  |
|  |  | 24.3 | High Arctic refugium for CAA: interglacial/glacial divergence | Eger et al., 2009; Klüstch et al., 2017 |  |  |  | 0.27 |  |
|  |  | 24.2 | High Arctic refugium for CAA: interglacial/glacial divergence and LGM admixture | Eger et al., 2009; Klüstch et al., 2017 | Gene flow from CAA into Mainland into CAA during LGM |  |  | 0.25 |  |
| NAAI26 (10,000 datasets) | Pop 1: CAA (including VIC)  Pop 2: EGBM  Pop 3: M  Pop 4: WG  Pop 5: BAFF | 26.1 | Holocene divergence | Gravlund et al., 1998; Flagstad et al., 2003 | BAFF most closely related to M | Scenarios 1 and 2 on either end of spread of scenario 3 simulated datasets. Observed data not closely overlapping simulated data. | Observed data not closely overlapping simulated data, closest to scenario 1 and 3. | 0.20 | 0.593 |
|  |  | 26.2 | Holocene divergence | Gravlund et al., 1998; Flagstad et al., 2003 | BAFF most closely related to CAA |  |  | 0.20 |  |
|  |  | 26.3 | Holocene divergence with CAA-M admixture | Gravlund et al., 1998; Flagstad et al., 2003 | BAFF formed from admixture between CAA and M |  |  | 0.29 |  |
| NAAI27a (10,000 datasets) | Pop 1: CAA Pop 2: EGBM Pop3: M (including BAFF) Pop4: WG Pop 5: VIC | 27.1 | Holocene divergences | Gravlund et al .,1998; Flagstad et al., 2003 | VIC most closely related to M | Scenarios 3 and 4 not distinct, scenario 2 and scenario 1 at opposite ends of spread of scenarios 3 and 4. Observed data not closely overlapping simulated data. | Observed data closest to simulated data in scenarios 3 and 4. | 0.32 |  |
|  |  | 27.2 |  | Gravlund et al .,1998; Flagstad et al., 2003 | VIC most closely related to CAA |  |  | 0.31 |  |
|  |  | 27.3 |  | Gravlund et al .,1998; Flagstad et al., 2003; McFarlane et al., 2016 | VIC formed from admixture between CAA and M |  |  | 0.56 |  |
|  |  | 27.4 |  | McFarlane et al., 2016 | VIC separate population most closely related to CAA, introgression into VI from M |  |  | 0.59 | 0.44 |
| NAAI27b (10,000 datasets) | Pop 1: CAA Pop 2: EGBM Pop3: M (including BAFF) Pop4: WG Pop 5: VIC | 27b.1 | Holocene divergences |  | Models the same as 27a without scenario 4 | Scenarios 1 and 2 distinct. Observed data closest to scenario 3. | Observed data overlapping simulated data in scenario 3. | 0.20 |  |
|  |  | 27b.2 |  |  |  |  |  | 0.22 |  |
|  |  | 27b.3 |  |  |  |  |  | 0.30 | 0.50 |
| NAAI27c (10,000 datasets) | Pop 1: CAA Pop 2: EGBM Pop3: M (including BAFF) Pop4: WG Pop 5: VIC | 27c.1 | Holocene divergences |  | Models the same as 27b except with broader admixture and VIC Ne priors | Scenarios 1 and 2 distinct. Observed data closest to scenario 3. | Observed data overlapping simulated data in scenario 3. | 0.16 |  |
|  |  | 27c.2 |  |  |  |  |  | 0.17 |  |
|  |  | 27c.3 |  |  |  |  |  | 0.26 | 0.60 |
| NAA27d (10,000 datasets) | Pop 1: CAA Pop 2: EGBM Pop3: M (including BAFF) Pop4: WG Pop 5: VIC | 27d.1 | Holocene divergences |  | Models the same as 27c except with broader admixture and VIC Ne priors, and additional founding population Ne parameter in 27d.3. | Scenarios 1 and 2 distinct. Observed data closest to scenario 3. | Observed data overlapping simulated data in scenario 3. | 0.15 |  |
|  |  | 27d.2 |  |  |  |  |  | 0.16 |  |
|  |  | 27d.3 |  |  |  |  |  | 0.23 | 0.62 |
| NAAI27e  (100,000 datasets) | Pop 1: CAA Pop 2: EGBM Pop3: M (including BAFF) Pop4: WG Pop 5: VIC | 27e.1 |  |  | Models the same as 27c except with separate ancestral Ne parameter. | Scenarios 1 and 2 distinct. Observed data closest to scenario 3. | Observed data overlapping simulated data in scenario 3. | 0.15 |  |
|  |  | 27e.2 |  |  |  |  |  | 0.16 |  |
|  |  | 27e.3 |  |  |  |  |  | 0.23 | 0.60 |
| NAAI25b (100,000 datasets) | Pop 1: CAA (including VIC)  Pop 2: EGBM  Pop 3: M (including BAFF)  Pop 4: WG | 25b.1 | Holocene divergence | Gravlund et al .,1998; Flagstad et al., 2003 | Same as NAAI25a except for scenario 2 where gene flow direction changed. | Observed data overlapping simulated data in scenarios 1 and 2. Scenarios 3 and 4 far from data and not distinct from each other. | Observed data overlapping simulated data in scenarios 1 and 2. | 0.35 |  |
|  |  | 25b.2 | Holocene divergence with CAA-M admixture | McFarlane et al., 2016 | Gene flow from M into CAA |  |  | 0.20 | 0.61 |
|  |  | 25b.3 | High Arctic refugium for CAA: interglacial/glacial divergence | Eger et al., 2009; Klüstch et al., 2017 |  |  |  | 0.301 |  |
|  |  | 25b.4 | High Arctic refugium for CAA: interglacial/glacial divergence and LGM admixture | Eger et al., 2009; Klüstch et al., 2017 | Gene flow from CAA into M into CAA during LGM |  |  | 0.29 |  |
| NAAI25c | Pop 1: CAA (including VIC) Pop 2: EGBM Pop3: M (including BAFF) Pop4: WG | 25c.1 |  |  | Same as NAAI25b except admixture parameter restricted between 0.1 and 0.75 as uninformative prior. | Observed data overlapping simulated data in scenarios 1 and 2. Scenarios 3 and 4 far from data and not distinct from each other. | Observed data overlapping simulated data in scenarios 1 and 2. | 0.33 |  |
|  |  | 25c.2 |  |  |  |  |  | 0.18 | 0.65 |
|  |  | 25c.3 |  |  |  |  |  | 0.29 |  |
|  |  | 25c.4 |  |  |  |  |  | 0.27 |  |
| NAAI25d | Pop 1: CAA (including VIC) Pop 2: EGBM Pop3: M (including BAFF) Pop4: WG | 25d.1 |  |  | Same as NAAI25b except with additional ancestral Ne parameter | Observed data overlapping simulated data in scenarios 1 and 2. Scenarios 3 and 4 far from data and not distinct from each other. | Observed data overlapping simulated data in scenarios 1 and 2. | 0.37 |  |
|  |  | 25d.2 |  |  |  |  |  | 0.22 | 0.71 |
|  |  | 25d.3 |  |  |  |  |  | 0.34 |  |
|  |  | 25d.4 |  |  |  |  |  | 0.34 |  |
| NAAI28 | Pop 1: WG  Pop 2: CAA (including VIC) Pop3: M (including BAFF) Pop4: EG | 26.1 |  |  | Changes from NAAI25d include new individuals: 2 WG, 4 M. Priors also constrained compared to NAAI25d. Note population numbering also different. | Observed data overlapping simulated data in scenarios 1 and 2. Scenarios 3 and 4 far from data and not distinct from each other. | Observed data overlapping simulated data in scenarios 1 and 2. | 0.37 |  |
|  |  | 26.2 |  |  |  |  |  | 0.21 | 0.83 |
|  |  | 26.3 |  |  |  |  |  | 0.35 |  |
|  |  | 26.4 |  |  |  |  |  | 0.35 |  |

**Table A2.3** **Evolutionary scenarios for Rangifer tarandus tested with DIYABC RF for the Barents Sea islands (BSI) region.** Populations: SVAL = Svalbard, NR = northern Russia, WR = western Russia, NZ = Novaya Zemlya, ER = Eastern Russia. Model sets tested against each other using Random Forest model classification with DIYABC RF when the simulated datasets from the training set and from the observed dataset overlapped on the first two axes of a principal component analysis (PCA), as recommended in the DIYABC manual (Prior scenario checks). Model choice refers to the overlap between observed and simulated data for each scenario on linear discriminant analysis (LDA) axes. Model posterior error corresponds to prediction quality exactly at the position of the observed dataset, with lower values indicating lower error rates and therefore higher prediction quality. Posterior probability reflects the most plausible, or “best model” within the tested model set.

| **Model set** | **Pop groups** | **Model** | **Key events** | **Source** | **Notes** | **Prior checks (PCA)** | **Model choice (LDA)** | **Class error** | **Posterior probability of preferred scenario** |
| --- | --- | --- | --- | --- | --- | --- | --- | --- | --- |
| BSI1 | Pop 1: SVAL Pop 2: NR/WR/NZ Pop3: ER | 1.1 | All divergence times from present to interglacial (100 kyr BP) |  | BSI1-3 focus on placement of NZ in topology and potentially multiple refugia | Simulated data overlap observed data | NA (no models to compare) | NA | NA |
| BSI2 | Pop 1: SVAL Pop 2: NR/WR Pop3: NZ  Pop 4: ER | 2.1 | All divergence times from present to interglacial |  | Potentially multiple refugia | Simulated data overlap observed data | NA (no models to compare) | NA | NA |
| BSI3 | Pop 1: SVAL/NZ Pop 2: NR/WR Pop3: ER | 3.1 | All divergence times from present to interglacial |  | Potentially multiple refugia | Simulated data overlap observed data | NA (no models to compare) | NA | NA |
| BSI1a | Pop 1: SVAL/NZ Pop 2: WR Pop3: NR/ER | 1a.1 | All divergences constrained to last glacial maximum (LGM). | Flagstad & Røed, 2003; Kvie et al., 2016 | NZ grouped with SVAL. BSI1a-3a focussed on placement of NZ and effect of excluding ER. Shared Beringian refugia | Simulated data close to but not overlapping observed data | NA (no models to compare) | NA | NA |
| BSI1b | Pop 1: SVAL/NZ Pop 2: WR Pop3: NR | 1b.1 | All divergences constrained to last glacial maximum (LGM). | Flagstad & Røed, 2003; Kvie et al., 2016 | NZ grouped with SVAL. ER excluded. Shared Beringian refugia | Simulated data overlap observed data | NA (no models to compare) | NA | NA |
| BSI2a | Pop 1: SVAL Pop 2: WR/NZ Pop3: NR/ER | 2a.1 | All divergences constrained to last glacial maximum (LGM) | Flagstad & Røed, 2003 | NZ grouped with WR. Shared Beringian refugia | Simulated data overlap observed data | NA (no models to compare) | NA | NA |
| BSI2b | Pop 1: SVAL Pop 2: WR/NZ Pop3: NR | 2b.1 | All divergences constrained to last glacial maximum (LGM) | Flagstad & Røed, 2003 | ER excluded. Shared Beringian refugia | Simulated data overlap observed data | NA (no models to compare) | NA | NA |
| BSI3a | Pop 1: SVAL Pop 2: NZ Pop3: WR Pop 4: NR/ER | 3a.1 | SVAL-NZ divergence in Holocene. NZ-mainland divergence up to LGM. | Kvie et al., 2016 | BSI3a & 3b have narrower divergence time priors. Potentially two refugia | Simulated data overlap observed data. Scenarios not distinct. | Simulated data overlaps observed data Scenario 1 has two peaks. | 0.28 | NA |
|  |  | 3a.2 | SVAL-NZ divergence in mid Holocene. NZ-mainland divergence in Holocene |  | Narrower divergence time priors. Potentially two refugia |  |  | 0.34 | 0.64 |
| BSI3b | Pop 1: SVAL Pop 2: NZ Pop3: WR Pop 4: NR | 3b.1 | SVAL-NZ divergence in Holocene. NZ-mainland divergence up to LGM. | Kvie et al., 2016 | ER excluded. NR and WR have negative Fst. Potentially two refugia | Simulated data overlap observed data. Scenarios not distinct. | NA (due to negative FST) | NA | NA |
|  |  | 3b.2 | SVAL-NZ divergence in mid Holocene. NZ-mainland divergence in Holocene |  | Narrower divergence time priors. Potentially two refugia |  |  | NA | NA |
| BSI4 | Pop 1: SVAL Pop 2: NZ Pop3: WR Pop4: NR Pop 5: ER | 4.1 | SVAL-NZ divergence in mid Holocene. NZ-mainland divergence constrained up to LGM | Kvie et al., 2016 | NR and WR have negative Fst. Potentially two refugia | Simulated data overlap observed data. Scenarios not distinct. | Simulated data overlaps observed data but closer to scenario 2 peak | 0.33 | NA |
|  |  | 4.2 | SVAL-NZ divergence in mid Holocene. NZ-mainland divergence constrained up to interglacial (100 kyr BP) |  | Broader priors. Island populations potentially in separate refugium. |  |  | 0.33 | 0.62 |
| BS14a | Pop 1: SVAL Pop 2: NZ Pop3: WR Pop4: NR Pop 5: ER | 4a.1 | SVAL-NZ divergence in mid Holocene. NZ-mainland divergence constrained up to LGM | Kvie et al., 2016 | Same as BSI4.1 but with faster mutation rate (1x10-8-1x10-5). 2022_11_16 | Simulated data overlap observed data. Scenarios not distinct. | Simulated data overlaps observed data. | 0.32 | 0.59 |
|  |  | 4a.2 | SVAL-NZ divergence in mid Holocene. NZ-mainland divergence constrained up to interglacial (100 kyr BP) |  | Same as BSI4.2 but with faster mutation rate (1x10-8-1x10-5). 2022_11_16 |  |  | 0.28 | NA |
| BS14b | Pop 1: SVAL Pop 2: NZ Pop3: WR Pop4: NR Pop 5: ER | 4b.1 | All divergence times from present to interglacial (100 kyr BP) |  | Same as BSI4.1 but with broader time priors. Potentially multiple refugia. | Simulated data overlap observed data. Scenarios not distinct. | Missing files - will rerun |  |  |
|  |  | 4b.2 | All divergence times from present to interglacial (100 kyr BP) |  | Same as BSI4.2 but with broader time priors. Potentially multiple refugia. |  |  |  |  |
| BSI4c | Pop 1: SVAL Pop 2: NZ Pop3: WR Pop4: NR Pop 5: ER | 4c.1 | SVAL-NZ divergence in mid Holocene. NZ-mainland divergence constrained up to LGM | Kvie et al., 2016 | Same as BSI4 but exclude River Mezen population. No negative Fst values anymore. Potentially two refugia. | Simulated data overlap observed data. Scenarios not distinct. | Simulated data overlaps observed data but bimodal distributions | 0.37 | 0.60 |
|  |  | 4c.2 | SVAL-NZ divergence in mid Holocene. NZ-mainland divergence constrained up to interglacial. | PCoA; Fst | Island populations potentially in separate refugium. |  |  | 0.3 | NA |
| BSI4d | Pop 1: SVAL Pop 2: NZ Pop3: WR Pop4: NR Pop 5: ER | 4d.1 | SVAL-NZ divergence in mid Holocene. NZ-mainland divergence constrained up to LGM | UPGMA; Kvie et al., 2016 | Same as scenario BSI4c.1. River Mezen population excluded. Potentially two refugia. |  | Only simulated data from scenario 1 overlaps observed data | 0.074 | 0.78 |
|  |  | 4d.2 | SVAL-NZ divergence in mid Holocene. NZ-mainland divergence constrained up to LGM. SVAL more recently diverged from NR than WR. | PCoA; Fst | River Mezen population excluded. Potentially two refugia. |  |  | 0.091 | NA |
| BSI2 | Pop 1: SVAL Pop 2: WR Pop3: NZ  Pop 4: NR/ER | 2.1 | SVAL divergence constrained up to LGM. NZ-mainland divergence constrained up to interglacial. | UPGMA; Kvie et al., 2016 | Potentially multiple refugia. |  | Simulated data overlaps observed data but closer to scenario 2 peak | 0.15 | NA |
|  |  | 2.2 | Island populations diverge in Holocene. Mainland populations diverge post-LGM. | UPGMA; Kvie et al., 2016; Flagstad & Røed, 2003 | One Beringean refugium. |  |  | 0.18 | 0.70 |
| BSI5 | Pop 1: SVAL Pop 2: NZ Pop3: WR Pop4: NR Pop 5: ER | 5.1 | SVAL-NZ divergence in mid Holocene. NZ-mainland divergence constrained up to LGM | UPGMA; Kvie et al., 2016 | Same as 4d.1. River Mezen population excluded. Potentially two refugia. |  | Simulated data from scenarios 3 and 4 overlap observed data completely. Scenarios 3 and 4 not distinguishable | 0.25 | NA |
|  |  | 5.2 | SVAL-NZ divergence in mid Holocene. NZ-mainland divergence constrained up to LGM. SVAL more recently diverged from NR than WR. | PCoA; Fst | Same as 4d.2. River Mezen population excluded. Potentially two refugia. |  |  | 0.27 | NA |
|  |  | 5.3 | NR more recently diverged from WR than ER. SVAL-NZ divergence in mid Holocene. NZ-mainland divergence constrained up to LGM | Fst | River Mezen population excluded. Potentially two refugia. |  |  | 0.51 | NA |
|  |  | 5.4 | NR more recently diverged from WR than ER; introgression from NR to NZ after SVAL divergence | Fst; PCoA | River Mezen population excluded. Potentially two refugia. |  |  | 0.51 | 0.72 |
| BSI6 | Pop 1: SVAL Pop 2: NZ Pop3: WR Pop4: NR Pop 5: ER | 6.1 | SVAL-NZ divergence in mid Holocene. NZ-mainland divergence constrained up to LGM |  | Same as 5.1 | Scenarios distinct and observed data closest to scenario 3. | Simulated data from scenario 3 overlap observed data. | 0.15 | NA |
|  |  | 6.2 | SVAL more recently diverged from NR than WR. |  | Same as 5.2 |  |  | 0.17 | NA |
|  |  | 6.3 | NR more recently diverged from WR than ER; introgression from NR to NZ after SVAL divergence |  | Same as 5.4 |  |  | 0.15 | 0.98 |
| BSI7 | Pop 1: FJL  Pop 2: ER Pop 3: SVAL Pop 4: NZ Pop 5: WR Pop 6: NR | 6.1 | SVAL-FJL and FJL-NZ divergences in mid Holocene. NZ-mainland divergence constrained up to LGM |  | Differences from BSI6: Additional samples: 113 SVAL, 4 NZ, 1 WR, 1 ER, 3 Ancient FJL.  River Mezen population also included. Note population numbering also different. | Scenarios distinct and observed data closest to scenario 1. | Simulated data from scenario 1 overlap observed data. | 0.16 | 0.83 |
|  |  | 6.2 | SVAL more recently diverged from NR than WR. |  |  |  |  | 0.16 | NA |
|  |  | 6.3 | NR more recently diverged from WR than ER; introgression from NR to NZ after FJL divergence |  |  |  |  | 0.012 |  |

**Table A2.4 DIYABC RF model specifications of final Rangifer tarandus North American Arctic islands (NAAI) model set.**

| **Priors** | **Scenario 1** | **Scenario 2** | **Scenario 3** | **Scenario 4** |
| --- | --- | --- | --- | --- |
| **Population size priors** | WG N1 N UN[10000,50000,0.0,0.0]  CAA N2 N UN[2000,12500,0.0,0.0]  M N3 N UN[350000,540000,0.0,0.0]  EG N4 N UN[3000,5000,0.0,0.0]  Ancestral NA N UN[5000, 300000,0,0] | | | |
| **Bottleneck size** | N1b N UN[5,250,0.0,0.0]  N4b N UN[5,500,0.0,0.0] | | | |
| **Bottleneck duration** | db T UN[10,50,0.0,0.0]  db2 T UN[75,150,0.0,0.0] | | | |
| **Admixture rate** | r2 A UN[0.1,0.75,0,0]  r3 A UN[0.1,0.75,0,0] | | | |
| **Conditions** | T3>T2, T3>T1, T2>TINT, T1>TINT, T6>T5, T6>T4, TINT2>T8, TINT2>T7, T9>TINT2 | | | |
| **Locus description** | <M> [S] G1 135 | | | |
| **Mutation priors** | group G1 [S]  MEANMU UN[1.00E-8,1.00E-6,5E-9,2]  GAMMU GA[1.00E-8,1.00E-6,Mean_u,2]  MEANK1 UN[0.050,20,10,2]  GAMK1 GA[0.050,20,Mean_k1,2]  MEANK2 UN[0.050,20,10,2]  GAMK2 GA[0.050,20,Mean_k2,2]  MODEL HKY 10 2.00 | | | |
| **Topology** | N1 N2 N3 N4  0 sample 1  0 sample 2  0 sample 3  0 sample 4  T1-db VarNe 4 N4b  T1 merge 2 4  T2-db2 VarNe 1 N1b  T2 merge 3 1  T3 merge 3 2  T3 varNe 3 NA | N1 N2 N3 N4 N1 N6  0 sample 1  0 sample 2  0 sample 3  0 sample 4  TINT split 2 5 6 r2  TINT merge 3 6  T1-db VarNe 4 N4b  T1 merge 5 4  T2-db2 VarNe 1 N1b  T2 merge 3 1  T3 merge 3 5  T3 varNe 3 NA | N1 N2 N3 N4  0 sample 1  0 sample 2  0 sample 3  0 sample 4  T4-db VarNe 4 N4b  T4 merge 2 4  T5-db2 VarNe 1 N1b  T5 merge 3 1  T6 merge 3 2  T6 varNe 3 NA | N1 N2 N3 N4 N3 N6  0 sample 1  0 sample 2  0 sample 3  0 sample 4  T7-db VarNe 4 N4b  T7 merge 2 4  T8-db2 VarNe 1 N1b  T8 merge 3 1  TINT2 split 3 5 6 r3  TINT2 merge 2 6  T9 merge 5 2  T9 varNe 5 NA |
| **Historical parameter priors** | T1 T UN[142, 1500,0.0,0.0]  T2 T UN[142,1500,0.0,0.0]  T3 T UN[1000,2000,0.0,0.0] | T1 T UN[142, 1500,0.0,0.0]  T2 T UN[142,1500,0.0,0.0]  T3 T UN[1000,2000,0.0,0.0]  TINT T UN[142,1500,0.0,0.0] | T4 T UN[142, 3429,0.0,0.0]  T5 T UN[142, 3429,0.0,0.0]  T6 T UN[142, 14300,0.0,0.0]  TINT T UN[142,1500,0.0,0.0] | T7 T UN[142,1500,0,0]  T8 T UN[142,1500,0,0]  TINT2 T UN[3000,14300,0,0]  T9 T UN[4300,14300,0,0] |

**Table A2.5 DIYABC RF model specification for the final Rangifer tarandus Barents Sea islands (BSI) model set.**

| **Priors** | **Scenario 1** | **Scenario 2** | **Scenario 3** |
| --- | --- | --- | --- |
| **Population size priors** | N1 N UN[750, 1500,0,0]  N2 N UN[25000, 150000,0,0]  N3 N UN[5000, 16000,0,0]  N4 N UN[1250, 3750,0,0]  N5 N UN[10000, 40000,0,0]  N6 N UN[50000,500000,0,0] | | |
| **Bottleneck size** | N4b N UN[5,500,0,0]  N8b N UN[5,500,0,0] | | |
| **Bottleneck duration** | db T UN[14,142,0,0] | | |
| **Admixture rate** | r2 A UN[0.01,0.5,0,0] | | |
| **Conditions** | t2>t1, t3>t2, t4>t3, t6>t5, t7>t6, t8>t7, t9>TINT, t10>t9, t11>t10, t12>t11 | | |
| **Locus description** | Locus_S_M_20_ <M> [S] G1 204 | | |
| **Mutation priors** | group G1 [S]  MEANMU UN[1e-08,1e-06,0,0]  GAMMU GA[1e-08,1e-06,Mean_u,2]  MEANK1 UN[0.05,20,0,0]  GAMK1 GA[0.05,20,Mean_k1,2]  MEANK2 UN[0.05,20,10,2]  GAMK2 GA[0.05,20,Mean_k2,2]  MODEL HKY 10 2 | | |
| **Topology** | N1 N2 N3 N4 N5 N6  0 sample 2  0 sample 3  0 sample 4  0 sample 5  0 sample 6  502 sample 1  t0 merge 1 3  t1 merge 4 1  t2-db VarNe 4 N4b  t2 merge 5 4  t3 merge 2 6  t4 merge 2 5 | N1 N2 N3 N4 N5 N6  0 sample 2  0 sample 3  0 sample 4  0 sample 5  0 sample 6  502 sample 1  t05 merge 1 3  t5 merge 4 1  t6-db VarNe 4 N4b  t6 merge 6 4  t7 merge 2 6  t8 merge 2 5 | N1 N2 N3 N4 N5 N6 N4 N4  0 sample 6  0 sample 2  0 sample 3  0 sample 4  0 sample 5  502 sample 1  t09 merge 1 3  TINT split 4 7 8 r2  TINT merge 6 7  t9 merge 8 1  t10-db VarNe 8 N8b  t10 merge 5 8  t11 merge 6 5  t12 merge 2 6 |
| **Historical parameter priors** | t0 T UN[502,1570,0,0]  t1 T UN[503,1570,0,0]  t2 T UN[503,3400,0,0]  db T UN[14,142,0,0]  t3 T UN[503,7000,0,0]  t4 T UN[503,14400,0,0] | t05 T UN[502,1570,0,0]  t5 T UN[503,1570,0,0]  t6 T UN[503,3400,0,0]  t7 T UN[503,14300,0,0]  t8 T UN[503,14400,0,0] | t09 T UN[502,1250,0,0]  TINT T UN[503,1250,0,0]  t9 T UN[503,1570,0,0]  t10 T UN[503,2500,0,0]  t11 T UN[503,3400,0,0]  t12 T UN[503,16000,0,0] |

# Appendix 3: Paleoclimate model specifications

Paleo-sea ice concentration was estimated from simulations using the global HadCM3B coupled atmosphere-ocean general circulation model with dynamic paleovegetation (specifically HadCM3LB-M2.1aD; Singarayer et al., 2017; Valdes et al., 2017). The atmospheric component model comprises a resolution of 2.5° x 3.75° latitude by longitude with 19 vertical levels, and a 30-minute timestep. The ocean component of the model has a horizontal resolution of 1.25°x 1.25° and 20 vertical levels, and a 1-hour timestep. Sea ice calculated as a zero-layer model on top of the ocean grid. The sea ice model uses a thermodynamic scheme and contains parameterizations of ice drift and leads. Sea ice cover is the fraction of the grid cell area covered by sea ice, which can take values up to 0.995, corresponding to a concentration measure of 99.5%. A detailed description of HadCM3LB-M2.1aD and the sea ice component model is available in Valdes et al. (2017). The MOSES2.1 land surface scheme is used, simulating water and energy fluxes and physiological processes. Fractional coverage of five plant functional types (PFTs; deciduous and needleleaf trees, C3 and C4 grasses, shrubs, with the residual assigned to bare soil) are incorporated by MOSES 2.1 and simulated by the dynamic global vegetation model (DGVM). This scheme has been shown to accurately reconstruct latitudinal variations in vegetation cover associated with large-scale changes in energy fluxes arising from orbital variability (Armstrong et al., 2023). The simulations have been forced with well-constrained orbital parameters (Berger et al., 1998) and greenhouse gas concentrations (CO_2_, N_2_O, and CH_4_) from the Vostok ice core (Loulergue et al., 2008; Spahni et al., 2005). When compared with observational datasets, the model accurately represents many aspects of the climate system on global and regional scales, including sea surface temperatures and ocean circulation (Valdes et al., 2017). For the paleoclimate simulations, sea-ice cover compares well with estimates of ice extent from microfossil data in regions with available data (Roche et al., 2012; Singarayer & Valdes, 2010).

The simulations use the ICE-5G glacial ice sheet model (Peltier, 2004), which includes detailed evolution of ice sheet thickness, extent, and continental isostatic rebound from the LGM to the present (1950 AD) at 500-year intervals. Continental ice sheets are included as a model boundary condition and are static through each individual model simulation. From this, total continental elevation, ice sheet extent, and land sea mask for each time interval were obtained.

# Appendix 4: DIYABC RF model choice output for final model sets

**Table A4.3 Model error rates for the final DIYABC RF model sets.** Global (prior) and scenario-specific error rates and RF classification votes for model choice for the final scenario set for the North American Arctic islands model (NAAI) and the Barents Sea islands model (BSI).

|  | **NAAI model** | | | | **BSI model** | | |
| --- | --- | --- | --- | --- | --- | --- | --- |
|  | **Scenario 1** | **Scenario 2** | **Scenario 3** | **Scenario 4** | **Scenario 1** | **Scenario 2** | **Scenario 3** |
| Prior error rate | 0.323 | 0.323 | 0.323 | 0.323 | 0.011 | 0.011 | 0.011 |
| Votes | 178 | 798 | 10 | 14 | 428 | 244 | 328 |
| Class error rate | 0.386 | 0.204 | 0.353 | 0.346 | 0.161 | 0.164 | 0.012 |

# Appendix A5: Genetic summary statistics of sampling populations

**Table A5.1 Genetic summary statistics of North American Arctic islands (NAAI) Rangifer tarandus populations.** Populations are given as the original population, herd, or locality based on sampling locality or region that was used in the original datasets/publications.

| **Population name** | **Number of individuals** | **Number of haplotypes** | **Gene diversity** | **Rarefied allelic richness** |
| --- | --- | --- | --- | --- |
| West Greenland | 16 | 1 | 0.5 | 1.77 |
| Bathurst Island | 12 | 4 | 0.754 | 2.36 |
| Bluenose East | 22 | 13 | 0.858 | 2.6 |
| Peary Islands | 12 | 4 | 0.686 | 2.21 |
| Banks Island | 32 | 11 | 0.795 | 2.45 |
| Victoria Island | 16 | 9 | 0.771 | 2.4 |
| Prince of Wales Island | 7 | 2 | 0.643 | 2.12 |
| Eglinton Island | 6 | 4 | 0.733 | 2.34 |
| Melville Island | 7 | 4 | 0.679 | 2.22 |
| Somerset Island | 3 | 3 | 0.833 | 2.6 |
| Baffin Island | 13 | 6 | 0.772 | 2.39 |
| Prince Patrick Island | 3 | 3 | 0.833 | 2.6 |
| Bathurst | 20 | 11 | 0.81 | 2.49 |
| Ellesmere Island | 121 | 3 | 0.666 | 2.14 |
| Baker Lake | 8 | 6 | 0.875 | 2.66 |
| CAA | 15 | 4 | 0.705 | 2.24 |
| East Greenland | 4 | 1 | 0.5 | 1.86 |

**Table A5.2 Genetic summary statistics of the Barents Sea islands (BSI) Rangifer tarandus populations.** Populations are given as the original population, herd, or locality based on sampling locality or region that was used in the original datasets/publications.

| **Population name** | **Number of individuals** | **Number of haplotypes** | **Gene diversity** | **Rarefied allelic richness** |
| --- | --- | --- | --- | --- |
| Franz Josef Land | 3 | 3 | 0.833 | 2.6 |
| Lake Chitta Region | 11 | 7 | 0.804 | 2.48 |
| Svalbard | 171 | 6 | 0.629 | 2.04 |
| Novaya Zemlya | 22 | 6 | 0.771 | 2.38 |
| Mezen River | 15 | 6 | 0.771 | 2.4 |
| Sakha Republic | 20 | 9 | 0.504 | 1.84 |
| Belyi Island, Yamal Nenets | 21 | 6 | 0.832 | 2.54 |
| Taymyr Peninsula | 59 | 36 | 0.888 | 2.68 |
| Pechora River | 13 | 10 | 0.766 | 2.4 |

**Table A5.3 Genetic differentiation (Pairwise Nei’s G_ST_ [Nei, 1973]) between North American Arctic islands (NAAI) Rangifer tarandus populations.** Populations are given as the original population, herd, or locality based on sampling locality or region that was used in the original datasets/publications.

|  | Baffin Isl. | Baker Lake | Banks Isl. | Bathurst | Bathurst Isl. | Bluenose East | CAA | East Greenland | Eglinton Isl. | Ellesmere Isl. | Melville Isl. | Peary Islands | Prince of Wales Isl. | Prince Patrick Isl. | Somerset Isl. | Victoria Isl. | West Greenland |
| --- | --- | --- | --- | --- | --- | --- | --- | --- | --- | --- | --- | --- | --- | --- | --- | --- | --- |
| Baffin Island | 0.000 | 0.019 | 0.124 | 0.015 | 0.023 | 0.027 | 0.073 | 0.410 | 0.027 | 0.092 | 0.139 | 0.072 | 0.113 | 0.043 | 0.043 | 0.020 | 0.360 |
| Baker Lake | 0.019 | 0.000 | 0.104 | 0.014 | 0.050 | 0.020 | 0.077 | 0.381 | 0.046 | 0.095 | 0.125 | 0.076 | 0.123 | 0.046 | 0.046 | 0.032 | 0.345 |
| Banks Island | 0.124 | 0.104 | 0.000 | 0.102 | 0.106 | 0.073 | 0.029 | 0.167 | 0.110 | 0.055 | 0.006 | 0.031 | 0.074 | 0.088 | 0.047 | 0.101 | 0.447 |
| Bathurst | 0.015 | 0.014 | 0.102 | 0.000 | 0.045 | 0.014 | 0.065 | 0.370 | 0.029 | 0.083 | 0.120 | 0.064 | 0.106 | 0.026 | 0.035 | 0.017 | 0.321 |
| Bathurst Island | 0.023 | 0.050 | 0.106 | 0.045 | 0.000 | 0.058 | 0.027 | 0.356 | 0.003 | 0.069 | 0.115 | 0.024 | 0.030 | 0.062 | 0.009 | 0.016 | 0.494 |
| Bluenose East | 0.027 | 0.020 | 0.073 | 0.014 | 0.058 | 0.000 | 0.061 | 0.346 | 0.047 | 0.068 | 0.102 | 0.062 | 0.116 | 0.024 | 0.041 | 0.028 | 0.325 |
| CAA | 0.073 | 0.077 | 0.029 | 0.065 | 0.027 | 0.061 | 0.000 | 0.235 | 0.033 | 0.013 | 0.041 | -0.014 | 0.007 | 0.046 | 0.003 | 0.038 | 0.477 |
| East Greenland | 0.410 | 0.381 | 0.167 | 0.370 | 0.356 | 0.346 | 0.235 | 0.000 | 0.383 | 0.348 | 0.116 | 0.222 | 0.224 | 0.439 | 0.262 | 0.386 | 1.000 |
| Eglinton Island | 0.027 | 0.046 | 0.110 | 0.029 | 0.003 | 0.047 | 0.033 | 0.383 | 0.000 | 0.068 | 0.132 | 0.031 | 0.048 | 0.045 | 0.010 | 0.007 | 0.478 |
| Ellesmere Island | 0.092 | 0.095 | 0.055 | 0.083 | 0.069 | 0.068 | 0.013 | 0.348 | 0.068 | 0.000 | 0.087 | 0.019 | 0.084 | 0.023 | 0.051 | 0.060 | 0.498 |
| Melville Island | 0.139 | 0.125 | 0.006 | 0.120 | 0.115 | 0.102 | 0.041 | 0.116 | 0.132 | 0.087 | 0.000 | 0.037 | 0.063 | 0.135 | 0.054 | 0.132 | 0.453 |
| Peary Islands | 0.072 | 0.076 | 0.031 | 0.064 | 0.024 | 0.062 | -0.014 | 0.222 | 0.031 | 0.019 | 0.037 | 0.000 | -0.001 | 0.051 | -0.001 | 0.043 | 0.478 |
| Prince of Wales Island | 0.113 | 0.123 | 0.074 | 0.106 | 0.030 | 0.116 | 0.007 | 0.224 | 0.048 | 0.084 | 0.063 | -0.001 | 0.000 | 0.133 | 0.008 | 0.080 | 0.590 |
| Prince Patrick Island | 0.043 | 0.046 | 0.088 | 0.026 | 0.062 | 0.024 | 0.046 | 0.439 | 0.045 | 0.023 | 0.135 | 0.051 | 0.133 | 0.000 | 0.053 | 0.024 | 0.460 |
| Somerset Island | 0.043 | 0.046 | 0.047 | 0.035 | 0.009 | 0.041 | 0.003 | 0.262 | 0.010 | 0.051 | 0.054 | -0.001 | 0.008 | 0.053 | 0.000 | 0.024 | 0.460 |
| Victoria Island | 0.020 | 0.032 | 0.101 | 0.017 | 0.016 | 0.028 | 0.038 | 0.386 | 0.007 | 0.060 | 0.132 | 0.043 | 0.080 | 0.024 | 0.024 | 0.000 | 0.434 |
| West Greenland | 0.360 | 0.345 | 0.447 | 0.321 | 0.494 | 0.325 | 0.477 | 1.000 | 0.478 | 0.498 | 0.453 | 0.478 | 0.590 | 0.460 | 0.460 | 0.434 | 0.000 |

**Table A5.4 Genetic differentiation (Pairwise Nei’s G_ST_ [Nei, 1973]) between Barents Sea islands (BSI) Rangifer tarandus populations**. Populations are given as the original population, herd, or locality based on sampling locality or region that was used in the original datasets/publications.

|  | Belyi Island, Yamal | Franz Josef Land | Chita-Baikal Region | Mezen River | Novaya Zemlya | Pechora River | Sakha Republic | Svalbard | Taymyr Peninsula |
| --- | --- | --- | --- | --- | --- | --- | --- | --- | --- |
| Belyi Island, Yamal | 0 | 0.113 | 0.119 | 0.12 | 0.137 | 0.074 | 0.148 | 0.21 | 0.045 |
| Franz Josef Land | 0.113 | 0 | 0.118 | 0.12 | 0.064 | 0.059 | 0.15 | 0.113 | 0.055 |
| Chita-Baikal Region | 0.119 | 0.118 | 0 | 0.126 | 0.167 | 0.08 | 0.152 | 0.216 | 0.063 |
| Mezen River | 0.12 | 0.12 | 0.126 | 0 | 0.166 | 0.054 | 0.155 | 0.218 | 0.068 |
| Novaya Zemlya | 0.137 | 0.064 | 0.167 | 0.166 | 0 | 0.095 | 0.196 | 0.083 | 0.097 |
| Pechora River | 0.074 | 0.059 | 0.08 | 0.054 | 0.095 | 0 | 0.107 | 0.144 | 0.026 |
| Sakha Republic | 0.148 | 0.15 | 0.152 | 0.155 | 0.196 | 0.107 | 0 | 0.25 | 0.088 |
| Svalbard | 0.21 | 0.113 | 0.216 | 0.218 | 0.083 | 0.144 | 0.25 | 0 | 0.144 |
| Taymyr Peninsula | 0.045 | 0.055 | 0.063 | 0.068 | 0.097 | 0.026 | 0.088 | 0.144 | 0 |

# Appendix 6: Population genetic clustering results

**Figure A6.1 Hierarchical population genetic clustering of Rangifer tarandus for the Barents Sea islands (BSI) and neighbouring mainland populations.** Dendrogram of original sampling populations using a UPGMA hierarchical clustering method based on pairwise Euclidean distances, where populations that are genetically closer to each other cluster together. Colours indicate non-hierarchical K-means clusters (K=4) identified using the average silhouette method (n = 335).

**Figure A6.2** **Population genetic clustering of Rangifer tarandus for the Barents Sea islands (BSI) and neighbouring mainland populations.** Ordination plot of metric multidimensional scaling (mMDS) of original sampling populations. mMDS represents distances between populations with the lowest possible dimensional space; populations closer together are more similar than those further apart. Colours represent the K-means clusters (K=4) identified using the average silhouette method on the mMDS coordinates (n = 335).

# Appendix 7: Genetic summary statistics of DIYABC model populations

**Table A7.1 Genetic summary statistics of the North American Arctic islands (NAAI) Rangifer tarandus DIYABC populations.** Populations refer to those used in the final model set.

| **Population name** | **Number of individuals** | **Number of haplotypes** | **Rarefied allelic richness** | **Gene diversity** |
| --- | --- | --- | --- | --- |
| Canadian Arctic Archipelago | 195 | 14 | 5.18 | 0.696 |
| East Greenland & Banks Melville | 43 | 13 | 7.26 | 0.759 |
| Mainland | 63 | 24 | 8.25 | 0.831 |
| West Greenland | 16 | 1 | 2 | 0.5 |

**Table A7.2 Genetic summary statistics of the Barents Sea islands (BSI) Rangifer tarandus DIYABC populations**. Populations refer to those used in the final model set.

| **Population name** | **Number of individuals** | **Number of haplotypes** | **Rarefied allelic richness** | **Gene diversity** |
| --- | --- | --- | --- | --- |
| Svalbard | 171 | 6 | 2.04 | 0.629 |
| Novaya Zemlya | 22 | 6 | 2.38 | 0.771 |
| Western Russia | 28 | 14 | 2.41 | 0.775 |
| Northern Russia | 80 | 37 | 2.66 | 0.881 |
| Eastern Russia | 31 | 15 | 2.23 | 0.698 |
| Franz Josef Land | 3 | 3 | 2.6 | 0.833 |

**Table A7.3 Genetic differentiation (Pairwise Nei’s G_ST_ [Nei, 1973]) between North American Arctic islands (NAAI) Rangifer tarandus DIYABC populations.** Populations refer to those used in the final model set.

|  | **Canadian Arctic Archipelago** | **East Greenland & Banks Melville** | **Mainland** | **West Greenland** |
| --- | --- | --- | --- | --- |
| **Canadian Arctic Archipelago** | 0.000 | 0.062 | 0.051 | 0.467 |
| **East Greenland & Banks Melville** | 0.062 | 0.000 | 0.105 | 0.467 |
| **Mainland** | 0.051 | 0.105 | 0.000 | 0.316 |
| **West Greenland** | 0.467 | 0.467 | 0.316 | 0.000 |

**Table A7.4 Genetic differentiation (Pairwise Nei’s G_ST_ [Nei, 1973]) between Barents Sea islands (BSI) Rangifer tarandus DIYABC populations used in the study.** Populations refer to those used in the final model set.

|  | **Svalbard** | **Novaya Zemlya** | **Western Russia** | **Northern Russia** | **Eastern Russia** | **Franz Josef Land** |
| --- | --- | --- | --- | --- | --- | --- |
| **Svalbard** | 0.000 | 0.083 | 0.165 | 0.149 | 0.192 | 0.113 |
| **Novaya Zemlya** | 0.083 | 0.000 | 0.116 | 0.097 | 0.143 | 0.064 |
| **Western Russia** | 0.165 | 0.116 | 0.000 | 0.039 | 0.070 | 0.076 |
| **Northern Russia** | 0.149 | 0.097 | 0.039 | 0.000 | 0.049 | 0.059 |
| **Eastern Russia** | 0.192 | 0.143 | 0.070 | 0.049 | 0.000 | 0.097 |
| **Franz Josef Land** | 0.113 | 0.064 | 0.076 | 0.059 | 0.097 | 0.000 |

# Appendix 8: Parameter estimates from final DIYABC RF models

**Table A8.1 Parameter estimates for North American Arctic islands (NAAI) *Rangifer tarandus* model scenario 2**. Estimates for the timing of population events in generations and calendar years before present, assuming a reindeer generation time of seven years. Estimates for population size are given for diploids (i.e. census population), twice the haploid (i.e. mitochondrial DNA) population size in the models. Populations: CAA = Canadian Arctic Archipelago, EGBM = East Greenland & Banks Melville, M = Mainland, WG = West Greenland.

|  |  | **Time parameters** | | | | | |
| --- | --- | --- | --- | --- | --- | --- | --- |
| **Parameter** | **Event type** | | **Populations** | **Median estimate (generations)** | **95% confidence interval (generations)** | **Median estimate (years)** | **95% confidence interval (years)** |
| TINT | Introgression | | M into CAA | 301 | 154-749 | 2107 | 1078-5243 |
| T1 | Divergence | | CAA EG | 532 | 188-1311 | 3724 | 1316-9177 |
| T2 | Divergence | | M WG | 699 | 219-1448 | 4893 | 1533-10136 |
| T3 | Divergence | | CAA M | 1532 | 1064-1952 | 10724 | 7448-13664 |
| db | Bottleneck duration | | EG | 22 | 11-45 | 154 | 77-315 |
| db2 | Bottleneck duration | | WG | 115 | 82-147 | 805 | 574-1029 |
| T2-db | End of bottleneck | | WG | 584 | 72-1366 | 4088 | 504-9562 |
| T1-db | End of bottleneck | | EG | 510 | 177-1300 | 3570 | 1239-9100 |
|  |  | **Other parameters** | | | | | |
| **Parameter** | **Type** | | **Population** | **Median estimate** | | **95% confidence Interval** | |
| N1 | Population size | | WG | 58686 | | 23002-96348 | |
| N2 | Population size | | CAA | 16904 | | 7012-24364 | |
| N3 | Population size | | M | 902622 | | 716078-1065370 | |
| N4 | Population size | | EG | 8878 | | 6680-9918 | |
| N1b | Bottleneck population size | | WG | 198 | | 22-458 | |
| N4b | Bottleneck population size | | EG | 714 | | 248-992 | |
| R2 | Admixture rate | | M into CAA | 0.491 | | 0.143-0.730 | |
| mu | Mutation rate | | - | 7.01x10^-7^ | | 2.64x10^-7^-9.63x10^-7^ | |

**Table A8.2 Parameter estimates for Barents Sea islands *Rangifer tarandus* model scenario 1**. Estimates for the timing of population events in generations and calendar years before present, assuming a reindeer generation time of seven years. Estimates for population size are given for diploids (i.e. census population), twice the haploid (i.e. mitochondrial DNA) population size in the models. Populations: FJL = Franz Josef Land, SVAL = Svalbard, NR = Northern Russia, WR = Western Russia, NZ = Novaya Zemlya, ER = Eastern Russia.

|  |  | **Time parameters** | | | | |
| --- | --- | --- | --- | --- | --- | --- |
| **Parameter** | **Event type** | **Populations** | **Median estimate (generations)** | **95% confidence interval (generations)** | **Median estimate (years)** | **95% confidence interval (years)** |
| T0 | Divergence | SVAL FJL | 857 | 535-1368 | 5999 | 3745-9576 |
| T1 | Divergence | FJL NZ | 1110 | 609-1539 | 7770 | 4263-10773 |
| T2 | Divergence | NZ WR | 1467 | 682-2765 | 10269 | 4774-19355 |
| T3 | Divergence | NR ER | 3563 | 1600-6631 | 24941 | 11200-46417 |
| T4 | Divergence | WR ER | 6711 | 3028-13269 | 46977 | 21196-92883 |
| db | Bottleneck duration | NZ | 72 | 18-135 | 504 | 126-945 |
| T2-db | End of bottleneck | NZ | 1395 | 547-2747 | 9765 | 3829-19229 |
|  |  | **Other parameters** | | | | |
| **Parameter** | **Type** | **Population** | **Median estimate** | | **95% confidence interval** | |
| N1 | Population size | FJL | 2430 | | 12587-31120 | |
| N2 | Population size | ER | 155978 | | 2850-7204 | |
| N3 | Population size | SVAL | 21858 | | 25372-49076 | |
| N4 | Population size | NZ | 5302 | | 288792-970552 | |
| N5 | Population size | WR | 47760 | | 105373-475332 | |
| N6 | Population size | NR | 464462 | | 121606-946332 | |
| N4b | Bottleneck population size | NZ | 642 | | 172-976 | |
| mu | Mutation rate | - | 5.78x10^-7^ | | 2.69x190^-7^-9.47x10^-7^ | |

# Appendix 9: Reindeer population events and modelled sea ice change

**Figure A9.1** **Holocene *Rangifer tarandus* population divergence events and modelled sea ice changes in the North American Arctic islands (NAAI).** (**a**, **c**): Demographic events mapped with relevant present-day populations, modern land extent (light grey), a snapshot of modelled mean sea ice concentration (blue - white) and land extent (dark grey) at 11 ka (**a**) and 5 ka (**c**) and reconstructed ice sheets at 10.9 ka (**a**) and 4.5 ka (**c**) – the closest time steps to the corresponding population events. Divergence time estimates are the median parameter estimates from the most plausible evolutionary model (scenario two). The geographic centroid between two populations (solid circle), within which 100 random samples of modelled sea ice concentration at different radii (dotted circle) were extracted and averaged (one radius shown for clarity). (**b**, **d**): Modelled sea ice concentration with associated standard deviations in 1 ka intervals. Demographic event times (dashed lines) with associated 95% credible intervals (dotted lines). Map projection North Pole Lambert azimuthal equal-area Canada.

**Figure A9.2 Holocene Rangifer tarandus population divergence, admixture, and modelled sea ice changes in the North American Arctic islands (NAAI).** (**a**, **c**): Demographic events mapped with relevant present-day populations, modern land extent (light grey), a snapshot of modelled mean sea ice concentration (blue - white) and land extent (dark grey) at 3 ka (a) and 2 ka (**c**), and reconstructed ice sheets at 3.2 ka (**a**) and 2 ka (**c**) – the closest time steps to the corresponding population events. Divergence time estimates are the median parameter estimates from the most plausible evolutionary model (scenario two). The geographic centroid between two populations (solid circle), within which 100 random samples of modelled sea ice concentration at different radii (dotted circle) were extracted and averaged (one radius shown for clarity). (**a**) Divergence and (**c**) admixture time estimates mapped with relevant present-day populations. (**b**, **d**) Modelled sea ice concentration with associated standard deviations in 1 ka time intervals. Timing of demographic events (dashed lines) with associated 95% credible intervals (dotted lines). Map projection North Pole Lambert azimuthal equal-area Canada.

**Figure A9.3 Holocene Rangifer tarandus population divergence events and modelled sea ice changes in the Barents Sea islands system (BSI).** (**a**, **c**, **e**): Demographic events mapped with relevant present-day populations, modern land extent (light grey), a snapshot of modelled mean sea ice concentration (blue - white) and land extent (dark grey) at 10 ka (**a**), 8 ka (**c**) and 6 ka (**e**). Divergence time estimates are the median parameter estimates from the most plausible evolutionary model (scenario three). The geographic centroid between two populations (solid circle), within which 100 random samples of modelled sea ice concentration at different radii (dotted circle) were extracted and averaged (one radius shown for clarity). Modelled sea ice concentration with associated standard deviations in 1 ka intervals. Estimated population divergence time (dashed lines) with associated 95% credible intervals (dotted lines). Map projection North Pole Lambert azimuthal equal-area Russia.

# Appendix References

Armstrong, E., Tallavaara, M., Hopcroft, P. O., & Valdes, P. J. (2023). North African humid periods over the past 800,000 years. *Nature Communications*, *14*(1), 5549. <https://doi.org/10.1038/s41467-023-41219-4>

Berger, A., Loutre, M. F., & Gallée, H. (1998). Sensitivity of the LLN climate model to the astronomical and CO2 forcings over the last 200 ky. *Climate Dynamics*, *14*(9), 615–629. https://doi.org/10.1007/s003820050245

BQCMB. (2014). *Beverly and Qamanirjuaq Caribou Management Plan 2013-2022*.

Burnett, H. A., Bieker, V. C., Le Moullec, M., Peeters, B., Rosvold, J., Pedersen, Å. Ø., Dalén, L., Loe, L. E., Jensen, H., Hansen, B. B., & Martin, M. D. (2023). Contrasting genomic consequences of anthropogenic reintroduction and natural recolonization in high-arctic wild reindeer. *Evolutionary Applications*, *16*(9), 1531–1548. https://doi.org/10.1111/eva.13585

Collin, F.-D., Durif, G., Raynal, L., Lombaert, E., Gautier, M., Vitalis, R., Marin, J.-M., & Estoup, A. (2021). Extending approximate Bayesian computation with supervised machine learning to infer demographic history from genetic polymorphisms using DIYABC Random Forest. *Molecular Ecology Resources*, *21*(8), 2598–2613. https://doi.org/10.1111/1755-0998.13413

Cuyler, C. (2010). West Greenland caribou explosion: What happened? What about the future? *Rangifer*, *27*. https://doi.org/10.7557/2.27.4.347

Davison, T. (2016). *Technical Report on the Cape Bathurst, Bluenose-West, and Bluenose-East Barren-ground Caribou Herds* (p. 90). Environment and Natural Resources, Government of Northwest Territories.

Department of Environment, Government of Nunavut. (2013). *‘Working together for Baffin Island Caribou’ Workshop Report (August 2013)* (p. 21). Department of Environment, Government of Nunavut.

Dussex, N., Tørresen, O. K., Valk, T. van der, Moullec, M. L., Veiberg, V., Tooming-Klunderud, A., Skage, M., Garmann-Aarhus, B., Wood, J., Rasmussen, J. A., Pedersen, Å. Ø., Martin, S. L. F., Røed, K. H., Jakobsen, K. S., Dalén, L., Hansen, B. B., & Martin, M. D. (2023). Adaptation to the High-Arctic island environment despite long-term reduced genetic variation in Svalbard reindeer. *iScience*, *26*(10), Article 10. https://doi.org/10.1016/j.isci.2023.107811

Eger, J. L., Gunn, A., & Baker, A. J. (2009). Genetic diversity and history of Peary caribou (Rangifer tarandus) in North America. *Proceedings of the Caribou Genetics and Relationships Workshop*, 45–62.

Flagstad, Ø., & Røed, K. H. (2003). Refugial origins of reindeer (Rangifer tarandus L.) inferred from mitochondrial DNA sequences. *Evolution*, *57*(3), 658–670. https://doi.org/10.1111/j.0014-3820.2003.tb01557.x

Gravlund, P., Meldgaard, M., Pääbo, S., & Arctander, P. (1998). Polyphyletic Origin of the Small-Bodied, High-Arctic Subspecies of Tundra Reindeer (Rangifer tarandus). *Molecular Phylogenetics and Evolution*, *10*(2), 151–159. https://doi.org/10.1006/mpev.1998.0525

Gunn, A. (2016). *Rangifer tarandus. The IUCN Red List of Threatened Species 2016* (No. e. T29742A22167140) [Dataset]. International Union for Conservation of Nature. <https://doi.org/10.2305/IUCN.UK.2016-1.RLTS.T29742A22167140.en>

Håkansson, S. (1976) University of Lurid radiocarbon dates IX. *Radiocarbon,* 18. 290-320.

Hold, K., Lord, E., Brealey, J. C., Le Moullec, M., Bieker, V. C., Ellegaard, M. R., Rasmussen, J. A., Kellner, F. L., Guschanski, K., Yannic, G., Røed, K. H., Hansen, B. B., Dalén, L., Martin, M. D., & Dussex, N. (2024). Ancient reindeer mitogenomes reveal island-hopping colonisation of the Arctic archipelagos. *Scientific Reports*, *14*(1), Article 1. https://doi.org/10.1038/s41598-024-54296-2

Kaluskar, S., Blukacz-Richards, E. A., Johnson, C. A., Kim, D.-K., & Arhonditsis, G. (2020). Connecting the dots in databases of endangered species: A Bayesian hierarchical imputation strategy for missing Peary caribou (Rangifer tarandus pearyi) population data. *Ecological Complexity*, *43*, 100846. <https://doi.org/10.1016/j.ecocom.2020.100846>

Kellner, F. L., Le Moullec, M., Ellegaard, M. R., Rosvold, J., Peeters, B., Burnett, H. A., Pedersen, Å. Ø., Brealey, J. C., Dussex, N., Bieker, V. C., Hansen, B. B., & Martin, M. D. (2024). A palaeogenomic investigation of overharvest implications in an endemic wild reindeer subspecies. *Molecular Ecology*, *33*(5), Article 5. https://doi.org/10.1111/mec.17274

Klütsch, C. F. C., Manseau, M., Anderson, M., Sinkins, P., & Wilson, P. J. (2017). Evolutionary reconstruction supports the presence of a Pleistocene Arctic refugium for a large mammal species. *Journal of Biogeography*, *44*(12), 2729–2739. https://doi.org/10.1111/jbi.13090

Kuhn, T. S., McFarlane, K. A., Groves, P., Mooers, A. Ø., & Shapiro, B. (2010). Modern and ancient DNA reveal recent partial replacement of caribou in the southwest Yukon. *Molecular Ecology*, *19*(7), 1312–1323. https://doi.org/10.1111/j.1365-294X.2010.04565.x

Kvie, K. S., Heggenes, J., Anderson, D. G., Kholodova, M. V., Sipko, T., Mizin, I., & Røed, K. H. (2016). Colonizing the High Arctic: Mitochondrial DNA reveals common origin of Eurasian archipelagic reindeer (Rangifer tarandus). *PLOS ONE*, *11*(11), e0165237. https://doi.org/10.1371/journal.pone.0165237

Le Moullec, M., Pedersen, Å. Ø., Stien, A., Rosvold, J., & Hansen, B. B. (2019). A century of conservation: The ongoing recovery of Svalbard reindeer. *The Journal of Wildlife Management*, *83*(8), 1676–1686. https://doi.org/10.1002/jwmg.21761

Letts, B., Fulton, T. L., Stiller, M., Andrews, T. D., MacKay, G., Popko, R., & Shapiro, B. (2012). Ancient DNA reveals genetic continuity in mountain woodland caribou of the Mackenzie and Selwyn Mountains, Northwest Territories, Canada. *Arctic*, *65*, 80–94. JSTOR.

Loulergue, L., Schilt, A., Spahni, R., Masson-Delmotte, V., Blunier, T., Lemieux, B., Barnola, J.-M., Raynaud, D., Stocker, T. F., & Chappellaz, J. (2008). Orbital and millennial-scale features of atmospheric CH4 over the past 800,000 years. *Nature*, *453*(7193), 383–386. https://doi.org/10.1038/nature06950

McFarlane, K., Gunn, A., Campbell, M., Dumond, M., Adamczewski, J., & Wilson, G. (2016). Genetic diversity, structure and gene flow of migratory barren-ground caribou (Rangifer tarandus groenlandicus) in Canada. *Rangifer*, *36*(1), 1. https://doi.org/10.7557/2.36.1.3577

Nei, M. (1973). Analysis of Gene Diversity in Subdivided Populations. *Proceedings of the National Academy of Sciences*, *70*(12), 3321–3323. https://doi.org/10.1073/pnas.70.12.3321

Pedersen, Å. Ø., Paulsen, I. M. G., Albon, S., Arntsen, G. B., Hansen, B. B., Langvatn, R., Loe, L. E., Le Moullec, M., Overrein, Ø., Peeters, B., Ravolainen, V., Ropstad, E., Stien, A., Tyler, N. J. C., Veiberg, V., Wal, R. van der, Andersen, R., Beumer, L. T., Eischeid, I., … Trondrud, L. M. (2019). Svalbard reindeer (Rangifer tarandus platyrhynchus): A status report. In *58* [Report]. Norsk Polarinstitutt. <https://brage.npolar.no/npolar-xmlui/handle/11250/2629207>

Peltier, W. R. (2004). Global glacial isostasy and the surface of the ice-age earth: The ICE-5G (VM2) Model and GRACE. *Annual Review of Earth and Planetary Sciences*, *32*(1), 111–149. https://doi.org/10.1146/annurev.earth.32.082503.144359

Petersen, S. D., Manseau, M., & Wilson, P. J. (2010). Bottlenecks, isolation, and life at the northern range limit: Peary caribou on Ellesmere Island, Canada. *Journal of Mammalogy*, *91*(3), 698–711. https://doi.org/10.1644/09-MAMM-A-231.1

Poole, K., Gunn, A., Patterson, B., & Dumond, M. (2010). Sea Ice and migration of the Dolphin and Union caribou gerd in the Canadian Arctic: An uncertain future. *Arctic*, *63*, 414–428. <https://doi.org/10.14430/arctic3331>

Roche, D. M., Crosta, X., & Renssen, H. (2012). Evaluating Southern Ocean sea-ice for the Last Glacial Maximum and pre-industrial climates: PMIP-2 models and data evidence. *Quaternary Science Reviews*, *56*, 99–106. https://doi.org/10.1016/j.quascirev.2012.09.020

Røed, K. H., Flagstad, Ø., Nieminen, M., Holand, Ø., Dwyer, M. J., Røv, N., & Vilà, C. (2008). Genetic analyses reveal independent domestication origins of Eurasian reindeer. *Proceedings of the Royal Society B: Biological Sciences*, *275*(1645), 1849–1855. https://doi.org/10.1098/rspb.2008.0332

Røed, K. H., Kvie, K. S., Losey, R. J., Kosintsev, P. A., Hufthammer, A. K., Dwyer, M. J., Goncharov, V., Klokov, K. B., Arzyutov, D. V., Plekhanov, A., & Anderson, D. G. (2020). Temporal and structural genetic variation in reindeer (Rangifer tarandus) associated with the pastoral transition in Northwestern Siberia. *Ecology and Evolution*, *n/a*(n/a). <https://doi.org/10.1002/ece3.6314>

Singarayer, J. S., Valdes, P. J., & Roberts, W. H. G. (2017). Ocean dominated expansion and contraction of the late Quaternary tropical rainbelt. *Scientific Reports*, *7*(1), Article 1. <https://doi.org/10.1038/s41598-017-09816-8>

Spahni, R., Chappellaz, J., Stocker, T. F., Loulergue, L., Hausammann, G., Kawamura, K., Flückiger, J., Schwander, J., Raynaud, D., Masson-Delmotte, V., & Jouzel, J. (2005). Atmospheric Methane and Nitrous Oxide of the Late Pleistocene from Antarctic Ice Cores. *Science*, *310*(5752), 1317–1321. https://doi.org/10.1126/science.1120132

Taylor, R. S., Manseau, M., Horn, R. L., Keobouasone, S., Golding, G. B., & Wilson, P. J. (2020). The role of introgression and ecotypic parallelism in delineating intraspecific conservation units. *Molecular Ecology*, *29*(15), Article 15. <https://doi.org/10.1111/mec.15522>

Valdes, P. J., Armstrong, E., Badger, M. P. S., Bradshaw, C. D., Bragg, F., Crucifix, M., Davies-Barnard, T., Day, J. J., Farnsworth, A., Gordon, C., Hopcroft, P. O., Kennedy, A. T., Lord, N. S., Lunt, D. J., Marzocchi, A., Parry, L. M., Pope, V., Roberts, W. H. G., Stone, E. J., … Williams, J. H. T. (2017). The BRIDGE HadCM3 family of climate models: HadCM3@Bristol v1.0. *Geoscientific Model Development*, *10*(10), 3715–3743. https://doi.org/10.5194/gmd-10-3715-2017

Wekʼèezhìi Renewable Resources Board. (2023). *Northern Caribou Canada*. Northern Caribou

Yannic, G., Pellissier, L., Ortego, J., Lecomte, N., Couturier, S., Cuyler, C., Dussault, C., Hundertmark, K. J., Irvine, R. J., Jenkins, D. A., Kolpashikov, L., Mager, K., Musiani, M., Parker, K. L., Røed, K. H., Sipko, T., Þórisson, S. G., Weckworth, B. V., Guisan, A., … Côté, S. D. (2014). Genetic diversity in caribou linked to past and future climate change. *Nature Climate Change*, *4*(2), 132–137. https://doi.org/10.1038/nclimate2074
